# Integration and electrical evaluation of WS_2_ and MoS_2_ fets in a 300 mm pilot line

**DOI:** 10.1007/s44291-026-00164-4

**Published:** 2026-02-01

**Authors:** T Schram, Q Smets, A Opdebeeck, S Ghosh, B Groven, P Kumar, H M Medina, D Cott, J.-F deMarneffe, H Dongre, A Kruv, L Panarella, L F Pinotti, P Morin, G. S. Kar, C. J. Lockhart de la Rosa

**Affiliations:** 1 IMEC, Kapeldreef 75, Leuven, 3001 Belgium; 2 KU Leuven, Leuven, Belgium

## Abstract

2D materials have the potential to extend and augment the CMOS scaling roadmap. However, upscaling from lab-based demonstrators to 300 mm-compatible integration modules presents unique challenges. In this work, we address these challenges through a 2D FET process flow developed within imec’s 300 mm Si pilot line. FETs with monolayer 2D transition metal dichalcogenide (TMDC) channels are fabricated, and the flow serves as a research vehicle to advance 300 mm-compatible integration modules, including TMDC growth, transfer, cleaning, gate stack deposition, and contact optimization. Key integration challenges arising from the weakly van der Waals-bonded 2D materials are identified, and the motivation behind specific process choices is discussed in the context of fab-specific contamination control. The processing is followed by a rigorous electrical characterization with dedicated test structures to evaluate the FET performance metrics, gate stack scalability, device yield, variability, reliability and stability. This paper provides, for the first time, a comprehensive and systematic description of our 300 mm fab-compatible integration flow for planar 2D FETs, broken down into its individual process modules and supported by a full electrical evaluation.

**Supplementary Information:**

The online version contains supplementary material available at10.1007/s44291-026-00164-4.

## Introduction

 The potential impact of 2D transition metal dichalcogenides (TMDCs) on semiconducting technology has been abundantly demonstrated [1–4]. However, most research on 2D TMDC materials is still conducted in lab-scale environments, relying on exfoliated flakes or lab-scale synthetic films, manual transfer, lift-off and electron-beam lithography [4–6]. Such approaches enable the rapid evaluation of device and process concepts, and offer a higher flexibility within terms of materials and techniques. Nevertheless, transitioning from sample-level or small-wafer experiments to production-scale integration on 300 mm wafers, which is today’s standard, remains far from obvious. The growth and transfer processes of the 2D films must be compatible with 300 mm wafer processing, as well as the associated techniques and materials regularly used in the lab environment. Although e-beam lithography can, in principle, be applied for 300 mm production, its limited throughput prevents high-volume manufacturing. Likewise, lift-off processes are unsuitable, as they are often incompatible with the elevated temperatures of many atomic layer deposition (ALD) processes used in an advanced production environment for highly scaled devices.

For these reasons, research on 300 mm fab-compatible integration of 2D TMDC devices is essential. This enables the evaluation of key device aspects, including the gate stack, contacts and overall device architecture, within a more mature and industrially relevant environment. This, in turn, helps to uncover additional integration that these materials will face, as well as potential solutions to address them. As such, we provide a step-by-step description of a process flow aimed at making TMDC test devices that respect the constraints of a production environment, while enabling the evaluation of the performance impact of different process steps, but also their effects on variability and reliability. To accelerate the learning cycles, a planar single sheet device architecture is selected. Nevertheless, the acquired knowledge is also relevant for the fabrication of more advanced architectures, such as stacked gate-all-around (GAA) devices with TMDC channels.

Additionally, planar 2D TMDC based devices remain of interest for earlier technology nodes, serving as active components in the back end, as well as for more advanced applications such as backside-integrated CMOS [7]. In both cases, the TMDC based transistors are likely to be integrated alongside an existing Silicon-based front-end that already includes lower-level interconnects. Consequently, the integration of TMDC devices integration must be fully compatible with a 300 mm Si-based production line. The presence of interconnects poses constraints on the thermal budget available for the TMDC device integration. Compatibility with a 300 mm Si production line therefore requires the use of representative Si process tools, while respecting the more stringent contamination control enforced in such a fab.

At imec, there has been considerable work done towards the goal of integrating 300 mm fab-compatible 2D TMDC based devices. In 2017, a first version of the 300 mm 2D integration flow was introduced [8]. The channel materials consisted of low-quality WS_2_ deposited either by plasma-enhanced atomic layer deposition (PEALD) or by chemical vapor deposition (CVD) conversion from Si seeds. In 2018, significant performance improvements were reported [9] by replacing the channel by 2-inch MoS_2_ patches grown via metal-organic CVD (MOCVD) on sapphire, followed by transfer using the thermal release tape (TRT) method to 300 mm wafers. A SiO_2_ interlayer by molecular beam deposition (MBD) of Si was also introduced, together with the top gate configuration. In 2020, the monolithic WS_2_ channel was further improved through MOCVD [10], and an early version of the 300 mm transfer dry method was electrically evaluated [9, 10]. In 2021, the TMA soak method was introduced to form a more uniform Al_2_O_3_ interlayer [11]. In subsequent work, the method was applied to WS_2_ FETs, incorporating a TiN/W-based back-end integration [12]. A comparative study of the four gate configurations for the proposed planar device (global back gate, top gate, local back gate, and dual gate) was also presented, along with a variability analysis. In 2022, an analysis was presented on the challenges of 300 mm integration of 2D materials, in particular the channel growth, patterning, delamination and contact formation [13]. Later, an evaluation was done of the gate stack reliability [14], pseudo-CMOS ring-oscillators were demonstrated [15] and WSe_2_ channels were introduced for the first time [16]. Significant transfer yield improvements were obtained in 2024 by optimizing the dry transfer process [17]. Alternatively, collective die-to-wafer transfer was demonstrated in 2023, yielding high-mobility single-crystal MoS_2_ channels [18], deposited by an optimized MOCVD process [19]. Although the process flow and the electrical characterization capabilities have steadily matured, many process steps remain unpublished, and a comprehensive overview has not yet been presented in the literature.

This paper provides a coherent and systematic description of the imec baseline 300 mm integration flow for planar 2D FETs, broken down into its individual process modules. The design and integration challenges are analysed, and the 2D-specific process choices are motivated for each step.

Section 2.1 describes the process flow variants, and the possible combinations of modules. The formation of the gates, contacts and interconnects is covered in Sect. 2.2–2.4 and 2.9–2.12. Section 2.5 presents two channel types: WS_2_ grown monolithically on the 300 mm device wafers, or MoS_2_ obtained by templated growth and transfer. The transfer and plasma cleaning of residues are discussed in Sect. 2.6. The capping of the channels is addressed in Sect. 2.7.

This is followed by an electrical assessment of the fabricated test structures. Section 3.1 covers the operation of different device types, followed by mobility and contact resistance extraction from 4-point test structures in Sect. 3.2. Yield, variability and capacitive effective thickness (CET) are covered in Sect. 3.3–3.5. Finally, the device reliability and stability are covered in Sect. 3.6–3.7. Several topics are reported here for the first time, including yield assessment, lot-to-lot variability, CET evaluation using FETCAP test structures, and the device stability. All other topics have been updated with the latest process protocols and significantly expanded compared to earlier reports in the literature.

## Process flow

The process flow is 300 mm Si fab compatible and aims to integrate single-sheet uni-channel transistors with transition metal dichalcogenide (TMDC) channels. The flow is a research vehicle aimed at making TMDC test devices that respect the above constraints and allows evaluating process options, such as gate stack and contact variation. Considering the general-purpose use of the test vehicle and to shorten the learning cycles, the devices are integrated straight on a 300 mm Si wafer. The motivation for the choice of processes and the process limitations are described. In case of the possible introduction of TMDC devices in the BEOL or on the backside of the wafer, the thermal budget would have to be limited. In some of the integration options, the thermal budget exceeds the acceptable limits, but in that case a solution to circumvent it is identified and is being evaluated.

The development of the TMDC device process flow is, except for some specific TMDC transfer instances, entirely performed in a 300 mm pilot fab. During the execution of the process flow, 300 mm fab tools are used exclusively, and the wafers are processed in the Si-based imec pilot line, hence sharing the toolset with the Si processing. Dedicated inspection and contamination handling procedures were introduced to allow the shared Si and TMDC processing.

The targeted process flow has a thermal budget below 500 °C, except for the monolithic TMDC deposition itself, and should hence be compatible with a future hybrid integration in the back end of line on top of a Si-based front end. The use of monolithically deposited 300 mm MOCVD WS_2_ enables the development of the different process modules prior to the availability of high-quality 300 mm TMDC growth and transfer processes. The compatibility of the process flow with high-quality channels is tested with 2-inch MoS_2_ deposited on sapphire and transferred to the 300 mm device wafers. Similarly, the integration of full-300 mm WS_2_ transfer from a 300 mm Si/SiO_2_ wafer to 300 mm device wafers is demonstrated. The growth of p-type WSe_2_ channels and the flow compatibility was previously demonstrated [16] but is considered out of scope for this article.

### Process flow variants

Four different types of devices with unique gate configurations can be achieved by combining the different mask layers and process modules, as summarized in Table 1. The simplest device type has a wafer-level global back gate (GBG) configuration. The Si wafer bulk acts as the back gate and is shared among all devices on the wafer. The second type is the local back gate configuration (LBG) with back gate connection (BGC) and Via To Bottom (VIATB). Here, the BGC is formed by a well implantation in the Si wafer, and the VIATB provides a connection to a frontside contact pad. This way, a bias can be applied to the back gate of individual devices. The remaining two configurations are obtained by adding the top gate module, either in combination with the global back gate or the local back gate.

**Table 1:** Different device types are obtained by combining different mask layers and integration modules. The local back gate is available either in TiN or highly doped Si

Device type → Mask/module↓	Global back gate (GBG)	Top gate + global back gate (TG)	Local back gate (LBG)	Top gate + local back gate (TG+LBG)
Local Back Gate Connection (BGC)	Optional	Optional	1	1
Local Back Gate (LBG)	0	0	1	1
Channel deposition	WS_2_ monolithic deposition ** or** 300 mm WS_2_ dry transfer ** or** MoS_2_ collective die-to-wafer transfer			
Active area (ACT)	1	1	1	1
Contact (M0)	1	1	1	1
Top Gate (TG)	0	1	0	1
Via To Bottom (VIATB)	optional	optional	1	1
Via (V0)	1	1	1	1
M1	1	1	1	1

**Fig. 1:**
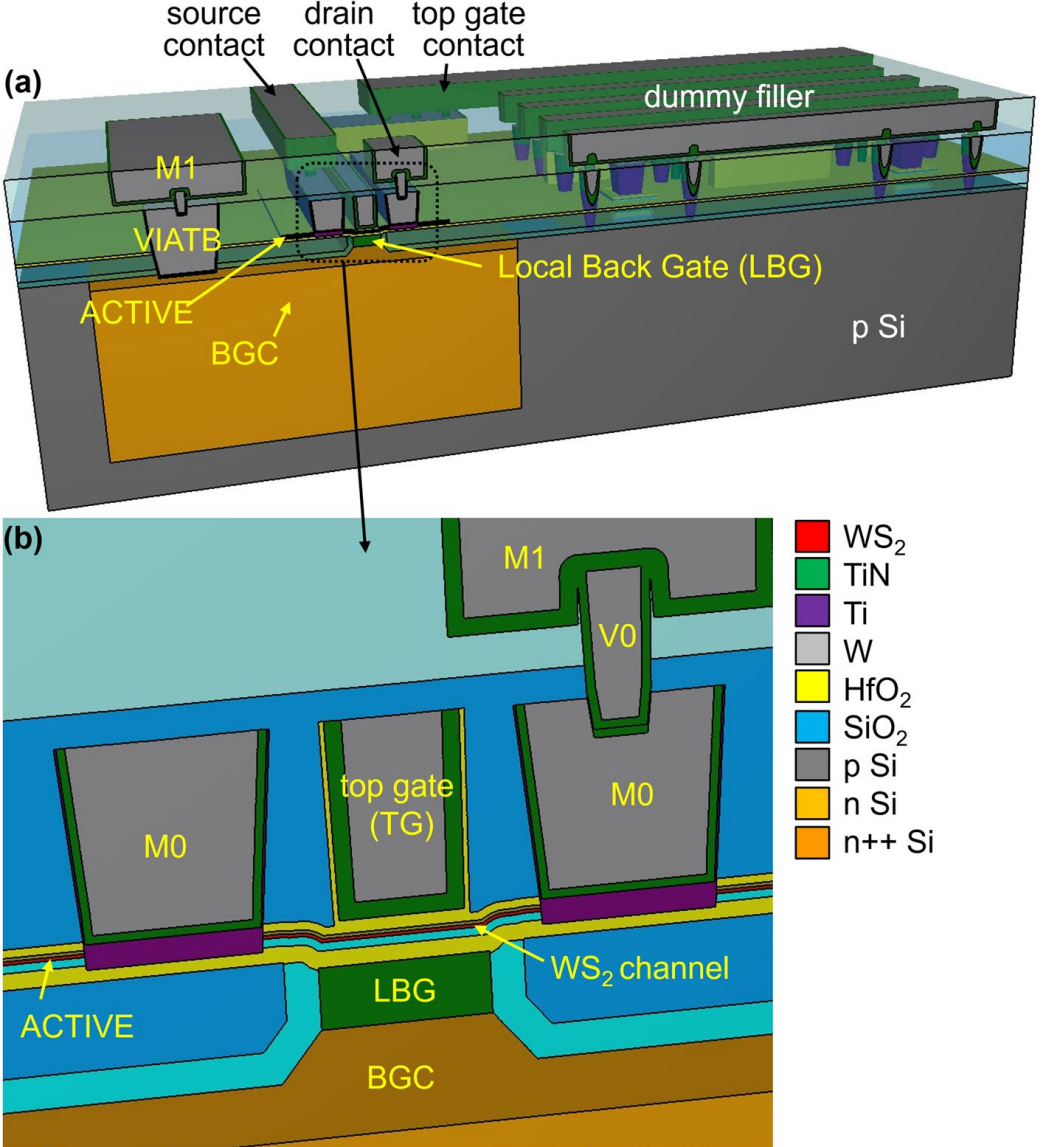
(**a**) 3D COVENTOR simulation of the final fully processed device with all process options and dummies included. (**b**) Zoom-in on the device region

**Fig. 2:**
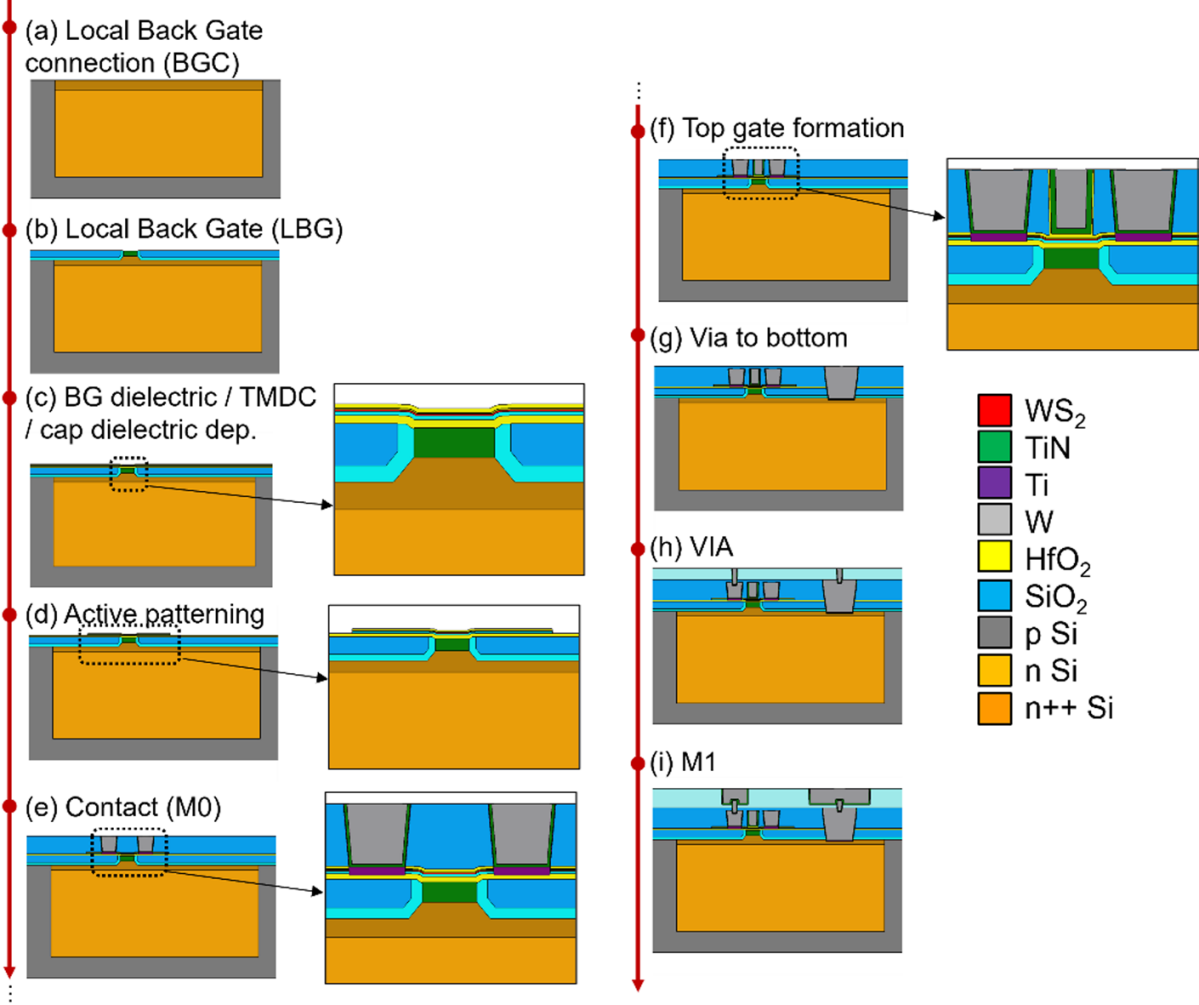
The process flow with individual process modules, as simulated by COVENTOR

A COVENTOR [20] based process simulation of the top gated + local back gated device (TG + LBG) is shown in Fig. 1(a-b). In this example, the local back gate is made of TiN. An alternative local back gate implementation with highly doped Si is also possible. The simulated area includes the semi-isolated device itself, and also a nearby dummy. These dummies are added to the masks to achieve a more uniform pattern density at all mask levels, but especially the levels that need chemical mechanical polishing (CMP) to guarantee within-die thickness uniformity. Figure 2 shows the device buildup as it proceeds through the different process modules, which are detailed in the following subsections.

### Local back gate connection

The purpose of the local back gate connection (BGC) module is to provide the electrical connection between the local back gate and the Via To Bottom (VIATB). Essentially, the BGC is a N-well formation in the P-type wafer, with a dopant concentration that increases to nearly degenerate N^+^ Si towards the surface. The nearly degenerate surface dopant concentration is used to ensure a good horizontal current conduction and the electrical connection to the metallic VIATB and LBG. The BGC wells have dimensions of at least 1 μm line width and spacing due to the combination of dopant diffusion and the lithographic limitation of the resist-based implant mask.

### Local back gate

**Fig. 3:**
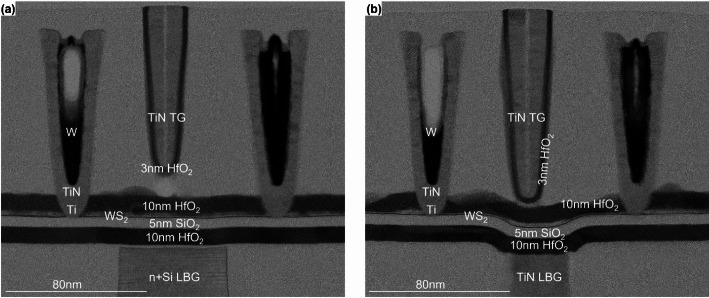
TEMs of device with (**a**) Si local back gate and (**b**) TiN local back gate. A slight corner rounding is observed in both cases after back gate dielectric deposition. Both devices have a WS_2_ channel deposited monolithically by MOCVD and are integrated with M0 contacts and a top gate. The vias and M1 are not yet processed

The local back gate (LBG) module is needed for the local back gated and dual gated device configurations. The local back gate can be made either from highly doped Si, or from TiN. Two important requirements which have guided the local back gate process choices are the minimization of surface roughness, and a step size of at most a few nm with the surrounding SiO_2_ to not jeopardize the subsequent TMDC transfer. A damascene (CMP) based approach would result in a gate surface with micro-roughness and scratches. Instead, an approach based on deposition, patterning and planarization approach is selected where the Si or TiN back gate surface is not affected by the CMP, and the as-deposited surface condition is maintained. The process steps for a TiN back gate are shown in the supplementary information (Figure S1). The TiN deposition method is selected to achieve minimal roughness. The step height at the gate edge is minimized by carefully tuning the dry etch steps, and results in 1 nm for Si and 5 nm for TiN local back gate as shown in Fig. 3.

The final step of the local back gate module is to deposit the back gate dielectric. This can be either 10 nm HfO_2_ by ALD (in case of MoS_2_ or WS_2_ channel transfer) or 10 nm HfO_2_ by ALD/5 nm SiO_2_ by PEALD (in case the WS_2_ channel is deposited monolithically).

### Global back gate

The global back gate configuration (GBG) is a simpler alternative to the local back gate configuration. The process starts with a Si wafer with n-type doping, followed by a degenerately n + surface implant, which acts as the back gate electrode. The global back gate insulator is either.

- 50 nm SiO_2_.
- or 50 nm SiO_2_/10 nm HfO_2_/5 nm SiO_2_.

**Fig. 4:**
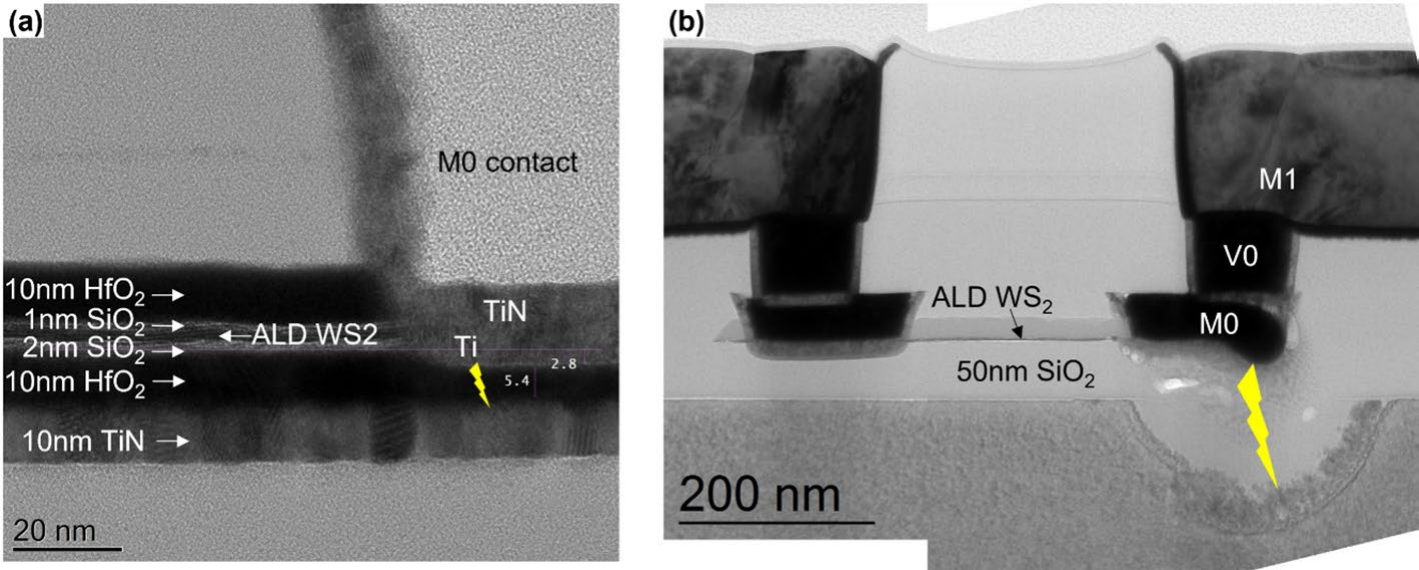
(**a**) A scaled global back gate dielectric 10 nm HfO_2_/2 nm SiO_2_ is affected by M0 over etching, causing dielectric breakdown during device operation and zero device yield. (**b**) A global back gate dielectric consisting of 50 nm SiO_2_ also has the risk of M0 over etching into the back gate oxide, causing a weak point below the M0 contacts and dielectric breakdown during device operation

The 50 nm SiO_2_ is made by thermal oxidation at 950 °C with a dry O2 process. This SiO_2_ is a high-quality and low-surface-roughness insulator, ideally suited for monolithic deposition of WS_2_ MOCVD. The 50 nm thickness is chosen to mitigate the impact of the M0 side contact overetch, which damages the back gate oxide. During earlier process development, a more scaled global back gate stack of 10 nm TiN/10 nm HfO_2_/2 nm SiO_2_ was attempted, but Fig. 4(a) shows that the M0 trench is over-etched by a few nanometers into the back gate oxide, causing dielectric breakdown during device operation. With a thicker 50 nm SiO_2_, excessive M0 trench over-etch can also cause dielectric breakdown when applying high voltages, as shown in Fig. 4(b). This varying over-etch is a source of variability and electrical yield loss (Sect. 3.3).

Therefore, the more complicated triple back oxide stack 50 nm SiO_2_ (thermal 950 °C)/10 nm HfO_2_ (ALD 300 °C)/5 nm SiO_2_ (PEALD 300 °C) offers higher integration robustness. The 10 nm HfO_2_ (ALD) acts as an etch stop layer for the ACTIVE, M0 and TG etch. The final 5 nm SiO_2_ (PEALD) serves as the dielectric interface for the monolithic channel deposition. The drawback of the more complicated triple stack is the possibly lower quality PEALD SiO_2_ as a growth dielectric, and the crystallization of HfO_2_ at high process temperatures causing a rougher growth surface and channel corrugation. In conclusion, both global back gate stacks have advantages and drawbacks, and the selection depends on the use case.

### Channel growth

The TMDC channels can be either monolayer WS_2_ or monolayer MoS_2_. Both are grown by metal-organic chemical vapor deposition (MOCVD), either on 300 mm Si/SiO_2_ wafers (monolithic) for the case of WS_2_, or on 2-inch sapphire wafers (template) for the case of MoS_2_. The purpose is to demonstrate two flows of production (monolithic growth, versus templated growth and transfer) and evaluate the transfer impact and yield in Sect. 3.3. The TMDC depositions are carried out in industry-compatible tools adapted from ASM crossflow epitaxial reactors. In both cases, the precursor chemistry consists of H_2_S and metal hexacarbonyl (M(CO)_6_) while nitrogen is selected as the carrier gas. For both channels, the chemical reaction stands as follows:

The typical process recipe includes an in-situ pretreatment at high temperature in a nitrogen or argon atmosphere, to prevent the presence of O_2_ in the chamber. The first deposition step aims to optimize nucleation at the wafer surface. During the second step, the crystals are grown laterally until coalescence and film closure, with process conditions minimizing parasitic nucleation both on the substrate and on top of the first monolayer. In the final stage, at the end of the deposition, the samples are annealed in a H_2_S-rich atmosphere to maximize crystallization and cooled to minimize the defect density in the same atmosphere [21]. After the deposition step, the wafers are either stored in N_2_ atmosphere and shielded from light until the encapsulation can be performed, to minimize the material degradation by photo-oxidation.

Subsection 2.5.1 discusses the monolithic deposition and characterization of WS_2_ on 300 mm Si/SiO_2_ wafers. Subsection 2.5.2 discusses the growth of MoS_2_ on a sapphire template, which must be followed by channel transfer, discussed in Sect. 2.6.

#### WS_2_ monolithic deposition

**Fig. 5:**
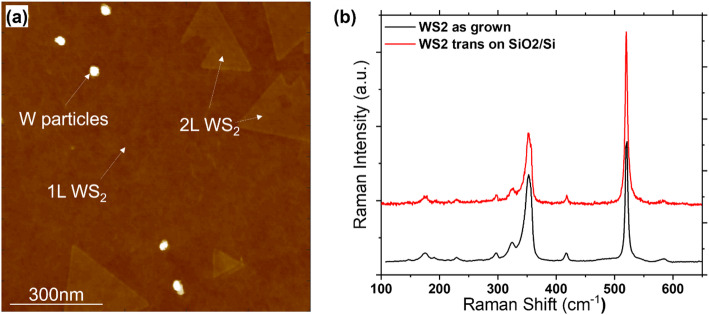
(**a**) AFM of WS_2_ with fully closed bottom layer, covered by second layer islands. (**b**) Raman intensity plot of WS_2_ after growth and after transfer (see Sect. 2.6.1)

The WS_2_ is deposited directly onto the 300 mm Si wafer, which already contains the global back gate or local back gate dielectric stack. Therefore, the topmost dielectric layer (either SiO_2_ by thermal oxidation, or SiO_2_ by PEALD) serves as the growth surface. We use a modified 300 mm epitaxial crossflow reactor (EPSILON3200, ASM). The metal precursor is W(CO)_6_ and the deposition temperature for the baseline WS_2_ channel is 950 °C. As the WS_2_ is deposited on amorphous SiO_2_, the WS_2_ crystals are randomly oriented, yielding to a polycrystalline material. Figure 5 pictures AFM images showing the presence of 2nd layer WS_2_ crystals on top of the closed polycrystalline first layer.

#### 1.1.1 MoS_2_ templated growth

To obtain a higher quality channel, MoS_2_ is deposited on 2-inch crystalline sacrificial sapphire substrates. The sapphire offers a crystallographic structure acting as a template, which allows the epitaxial registry of MoS_2_ and the formation of large monocrystalline domains.

We use a 200 mm crossflow reactor (Polygon8200 from ASM) modified to enable the MOCVD of MoS_2_. Pocket wafers enable the handling of the 2-inch sapphire wafers and allows the growth on two substrates at a time. First, the sapphire wafers are loaded into the load lock where a base pressure of ~ 1 mTorr is reached and then refilled with N_2_ before going into the chamber. This allows the chamber to be kept oxygen-free. For the standard deposition process, the samples are heated to 1000 ˚C in high-purity nitrogen. Afterwards, Mo(CO)_6_ and H_2_S are flown into the chamber for 25 min. The relative concentrations of precursors [H_2_S: Mo(CO)_6_] is 13k. The high vapor pressure of the sulphur precursor compared to the metal precursor is required to maintain the stoichiometry of the films at elevated temperatures. Then, the delivery of Mo(CO)_6_ is closed, and the samples are kept for 10 min at 1000 ˚C under H_2_S to allow the recrystallization of MoS_2_. The grown MoS_2_ is cooled down to 300 ˚C in H_2_S and Nitrogen atmosphere before unloading, in order to avoid oxidation.

**Fig. 6:**
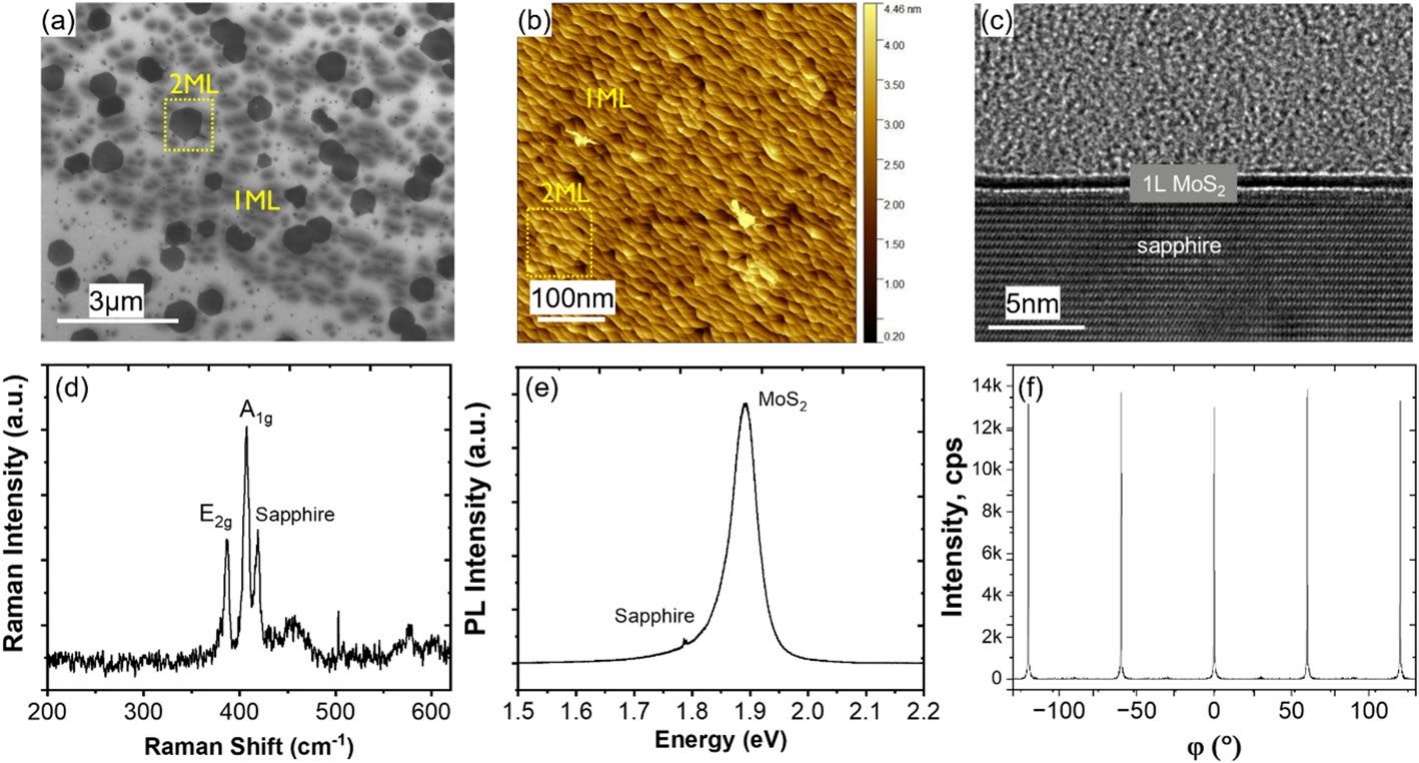
Top view (**a**) SEM, (**b**) AFM and (**c**) cross-sectional TEM of monolayer MoS_2_ grown on sapphire. (**d**) Raman, (**e**) PL and (**f**) grazing incidence in-plane XRD of the same material

Under these conditions, the grown MoS_2_ is mostly a monolayer, with 20% coverage of bilayer islands (Fig. 6(a-b)). Cross-section TEM presented in Fig. 6(c) reveals the monolayer nature of the film. This is furthermore supported by Raman spectroscopy, where the difference A_1g_ – E_2g_ is ~ 20 cm^− 1^, and by the strong photoluminescence response at 1.89 eV, as expected for the transition to a direct bandgap at the monolayer as shown in Fig. 6(d-e).

The epitaxial behaviour and crystallinity of the as-grown MoS_2_ film are revealed using grazing-incidence in-plane XRD (GIIXRD) in Fig. 6(f) showing only the 60° diffraction peaks on the φ-scan, as expected for single crystalline hexagonal material.

### Channel transfer

**Fig. 7:**
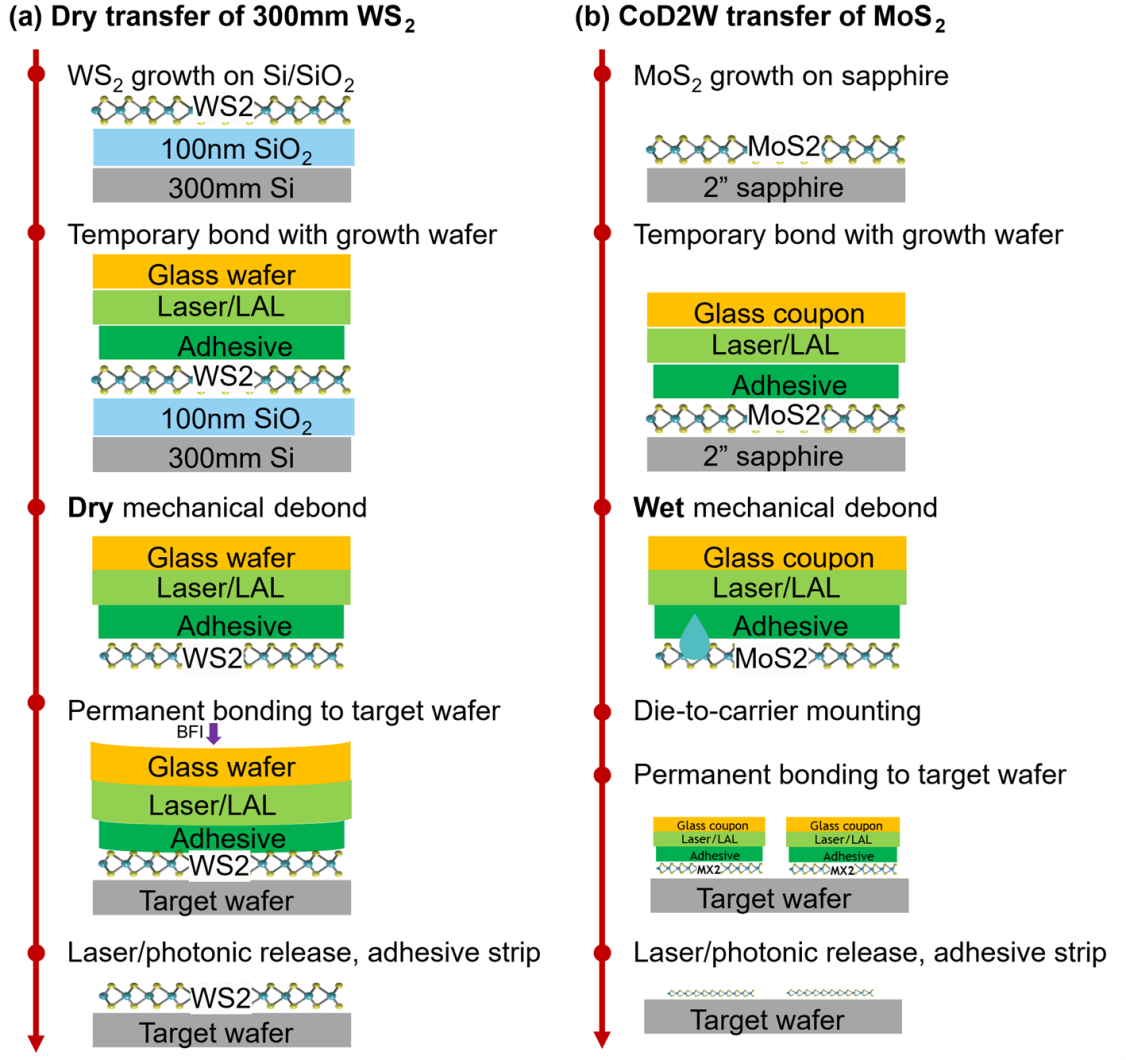
Overview of layer transfer module implemented for transferring material (**a**) grown on 300 mm Si and (**b**) 2 inch sapphire

Channel transfer strongly increases the flexibility of the process flow, because there is a decoupling between the TMDC growth (with harsh growth conditions and dedicated substrates), and the device wafers (with strict requirements on the thermal budget and on the bottom gate stack). Two transfer types are available, as summarized in Fig. 7. The first type is dry transfer of 300 mm-scale WS_2_. The second type is collective die-to-wafer (CoD2W) transfer, where several patches of TMDC are debonded from 2” sapphire substrates and collectively transferred to a 300 mm device wafer. The details of both methods follow below.

#### Dry transfer of WS_2_ grown on 300 mm Si/SiO_2_

When WS_2_ is grown on 300 mm Si covered by thermal SiO_2_, it can be dry transferred to new 300 mm device wafers, also called target wafers. This process was previously reported [17] and is described in greater detail below.

The process is initiated by bonding the growth wafer with WS_2_ to a thermoplastic adhesive (Brewer Science BrewerBond^®^ 305) and laser-releasable polymer coated glass carrier wafer, as shown in Fig. 7(a). The bonding is performed in a bonder at 10^− 2^ – 10^− 3^ Torr, 200 °C, and the applied force between the bonded wafers is 4500 N. The bonded stack is cooled in vacuum such that the temperature during unloading is less than the glass transition temperature (T_g_) of the adhesive. Mechanical debonding of this bonded stack is then performed by applying a controlled strain (orthogonal to the bonded plane) starting from the wafer-notch to the other end, such that the debond rate is between 0.5 and 1 cm/s, while keeping the glass carrier flat.

This is followed by permanent bonding to the target wafers. Initiating and controlling the bond wave at this step is crucial to enable reliable and reproducible TMD transfer. The process is executed in the following sequence: (1) The bond chamber is evacuated, and the two wafers are brought in proximity of 1 mm from each other. (2) A spring-loaded bond-pin initiates the contact between the wafers by bowing it downwards at the center of the top wafer, and the bond wave propagates from the center to wafer edge as the edges are relaxed, ensuring a uniform van der Waals contact at the interface. (3) The bond force is subsequently increased to 4500 N to anchor the wafers in place. (4) The temperature is ramped to 200 °C to allow for adhesive reflow, while mitigating any physical migration of the underlying TMD. (5) Finally the bonded stack is cooled down below T_g_ before removing the force, venting the chamber and unloading the stack.

At this point the bonded stack can be laser released by ablating the laser-release layer or debonded via photonic debonding (PulseForge^®^ PD300 SA) if the original stack had a light absorbent layer (typically metallic coating on glass). Finally solvent based residue and adhesive cleaning ensures a homogeneous transfer.

The above-described transfer process is described for WS_2_ grown on SiO_2_, but can be applied to most TMDs that are grown on amorphous substrates. Further, the transfer process has also been validated on target wafers with local back gates that present nominal local topography ranging from 3 to 7 nm, as discussed in Sect. 2.3. Our tests demonstrate reliable transfer yield at lower step heights (< 5 nm). In addition, edge rounding at the step also mitigates accumulated strain by a certain extent, however the bond-front propagation velocity becomes even more crucial to control for such substrates. For steps > 5 nm, our data suggests high propensity of crack formation and decrease in transfer yield after the permanent bonding step. Other than the permanent bonding step, alternate modes of transfer failure (usually at the dry-mechanical-debonding step) occur during three primary cases: (1) Presence of high density of particles due to parasitic metal formation during the TMDC growth, (2) high degree of vertical TMDC growth (poor vdW interface between TMD and growth oxide), and (3) high surface roughness of underlying amorphous oxide after growth.

#### Collective die to wafer (CoD2W) transfer

Forming single crystalline TMD requires templated growth on sapphire, as discussed in Sect. 2.5.2 for MoS_2_. The single crystalline sapphire surface allows growth with a higher degree of contiguity and with orientation control at multiple nucleation sites and subsequently a controlled coalescence of the grains. Thereby the relative vdW adherence between MoS_2_ and the sapphire substrate is relatively stronger than between WS_2_ and amorphous SiO_2_. Hence, additional stimulus is required during the mechanical debonding process, in the form of water intercalation. The transfer process was previously reported [18] and is described in greater detail below and depicted in Fig. 7(b).

The process is initiated with temporary bonding, very similar to that of 300 mm shown in Fig. 7(a). However, due to the higher adhesion between the TMD and sapphire, the bonded stack is immersed in warm-DIW at 60 °C (below T_g_ of adhesive) for 10 min, ensuring water intercalation at this interface. In literature, DIW is often replaced with stronger intercalating agents such as KOH, NaOH; however, for maintaining fab-processing specs, such reagents are not desired and subsequently necessitate extreme control on high quality growth to facilitate delamination.

The mechanical debonding is performed on a custom-made tool, which holds the sapphire on the bottom vacuum chuck. Next, a motorized z-directional stage controllably pushes the glass coupon upwards (away from the bond-plane, while a stationary roller affixed above the glass and parallel to the bond-plane keeps the bond wave from propagating to the wafer-end uncontrollably. This effectively bends the top glass wafer within a range of 4–5° relative to the bond-plane and creates an “initial crack” whereby the initial edge of the TMD is delaminated from sapphire. At this stage more water is injected at the interface (~ 1–2 mL) to ensure that the debond-wave propagation at the subsequent steps occurs in the visible presence of water at that interface. At this point, a linear actuator affixed to the stationary roller can initiate a controlled motion orthogonal to the Z-pusher and the initial crack line, and parallel to the bond-plane. The two actuators are programmed to translate such that an automated debonding occurs for the rest of the stack and the end debond angle is twice the angle at initial crack formation. This results in complete delamination of the carrier wafer from the underlying sapphire.

**Fig. 8:**
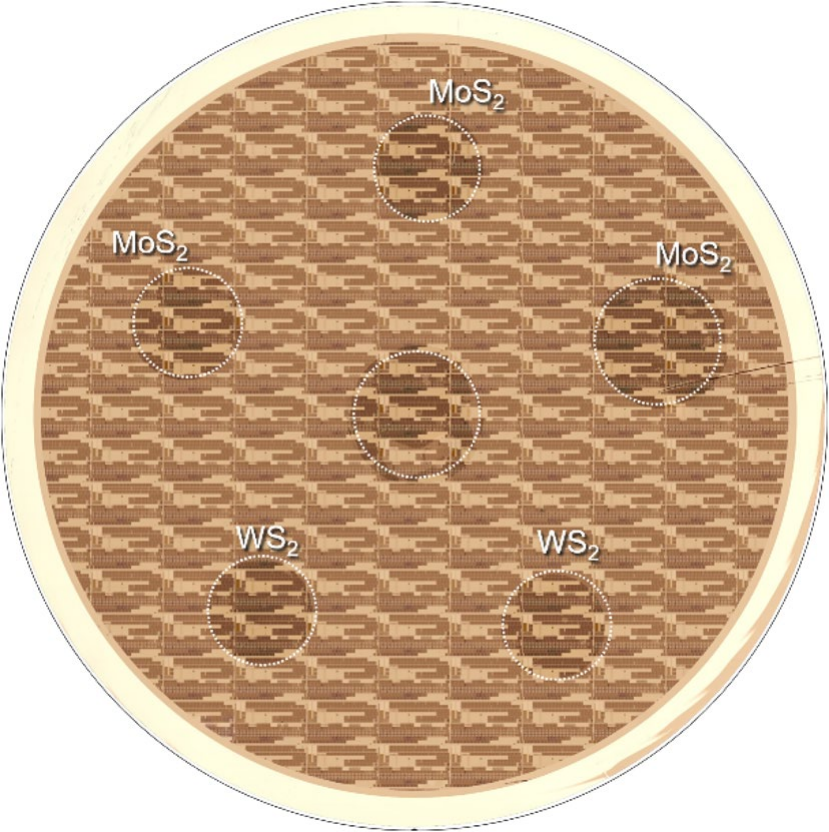
Optical image of a 300 mm wafer with patches of 2” MoS_2_ and WS_2_. The pattern seen is due to active patterning [18].

The permanent bonding step is done by placing the 300 mm target wafer on the bottom chuck of the bonder. The coupons (representing the “dies”) are aligned with a laser-cut template affixed above the bottom Si target wafer, thus evolving into a CoD2W transfer mechanism. The overall permanent bonding sequence following collective die-mounting, the subsequent unloading, release and solvent cleaning are identical to that described in Sect. 2.6.1. Figure 8 shows an example optical image after collective die-to-wafer transfer of MoS_2_ (grown on sapphire) and WS_2_ (grown on SiO_2_).

An alternative and widely used transfer technique is the thermal-release-tape (TRT) assisted lamination/delamination. This technique cannot be upscaled to larger dimensions for full-wafer transfer, but it is used in this work as a reference for the development of glass-based transfer methods. In the TRT method, a 2-inch sapphire substrate with MoS_2_ is spin coated with PMMA and baked to cure the adhesive. A pre-cut TRT is laminated on the PMMA/TMD/sapphire stack at 80 °C and immersed in DIW (60 °C) for 6–10 min where it delaminates during pick up with tweezers. This delaminated tape is N_2_ blow dried and laminated again on the 300 mm target at room temperature. During this lamination step (albeit dependent on operator finesse), it is imperative to minimize any encapsulated voids between the TMD and the target surface. This is further alleviated by using a squeegee to press out the bubbles. Subsequently, the TRT is delaminated from the target by heating the stack at 150 °C and finally the PMMA is removed with a hot acetone wash overnight.

#### Post-transfer residue clean

The use of solvents to clear the bulk PMMA film after TRT release is a widely accepted cleaning method. However, nanometric PMMA residues remain on the surface. They are difficult to remove by the solvent clean due to their specific conformation on the surface, which is described in literature as irreversibly physically adsorbed layer (IPAL). Due to the many surface anchoring points of the entangled polymer, the energy (thermal agitation) needed to reach full detachment cannot be achieved in liquids, even when warmed close to their boiling point and with high dilution power or aided by ultrasonication.

Our solution is to fully degrade the polymer by means of reactive plasma, leading to the formation of volatile hydrocarbon by-products (CH_4_ but generally described as C_x_H_y_O_z_). The description of the IPAL and the plasma cleaning is described in detail by Marinov et al. [22]. The PMMA dry cleaning process uses a highly diluted H_2_-He (1%) downstream plasma, in a 300 mm Microwave Stripper by Lam Research Corp., at a substrate temperature of 300 °C. To develop selectivity towards the MoS_2_ or WS_2_ monolayer, a reducing chemistry is preferred – H_2_. The high temperature enhances the degradation of the PMMA (thermally activated mechanism) but also diminishes the degradation of the MX_2_, which occurs through sulfur loss. Indeed, the formation of S vacancies require not only breaking the M-S chemical bond but also forming H_2_S (volatile). The formation of H_2_S requires a high density of mobile H* radicals on the surface, which is lowered due to the high H*+H* → H_2_ surface recombination rate at elevated temperature. Although it is known that a 300 °C annealing (without plasma exposure) can cause sulfur loss [23], the duration of our treatment remains sufficiently short (10 to 25 s) to avoid significant sulfur loss due to the thermal effect only.

**Fig. 9:**
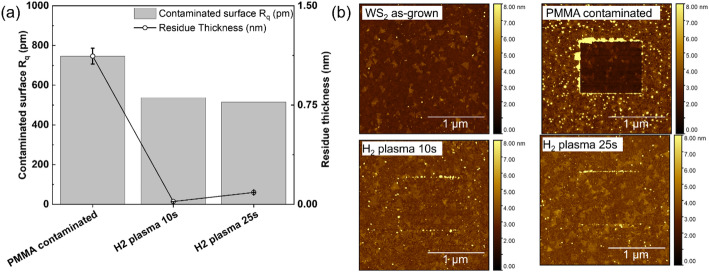
(**a**) Evolution of residues characteristics with H_2_ plasma clean. (**b**) The AFM images illustrate the surface morphology using the tip-cleaning method, where the central square area is mechanically cleaned by a secondary AFM tip, enabling the precise measurent of the PMMA residue thickness. The round white particles are other surface defects, not considered in this study

The effect of the plasma cleaning is apparent from Fig. 9(a-b), which shows the PMMA residue thickness and surface roughness (R_q_) as measured by AFM microscopy. The initial PMMA residue thickness is of the order of ~ 1.2 nm. After 10 s of He-H_2_ plasma, the PMMA thickness has been reduced to within measurement error, i.e. no more PMMA residues can be detected. This is correlated with the surface roughness R_q_, which decreases close to the pristine WS_2_ range 350–450 pm. The cleaning efficiency is confirmed visually on the AFM images in Fig. 9(b).

### Interlayer and cap dielectric deposition

The next step in the fabrication process is to deposit a 1 to 1.5 nm Al_2_O_3_ interlayer and a 5–10 nm HfO_2_ capping dielectric on the TMDC channel. The goal is to protect the channel during the active patterning and later processing steps, since monolayer MoS_2_ and WS_2_ have a high sensitivity to plasma-induced damage, oxidation in ambient air, and water-assisted delamination. The Al_2_O_3_ interlayer and HfO_2_ cap both become part of the top gate dielectric stack in top gated devices (Sect. 2.10).

The interlayer and cap are deposited in a close coupled fashion with the TMDC deposition. If the TMDC were to be exposed to the ambient, the adsorption of residues or airborne contaminants [24, 25] would hinder the cap deposition. Consequently, the time interval between the TMDC and the top oxide deposition is kept to a minimum. When delays are unavoidable, the wafers are stored in a black N2-purged front-opening unified pod (FOUP) to limit photo-induced oxidation in air ambient [11].

**Fig. 10:**
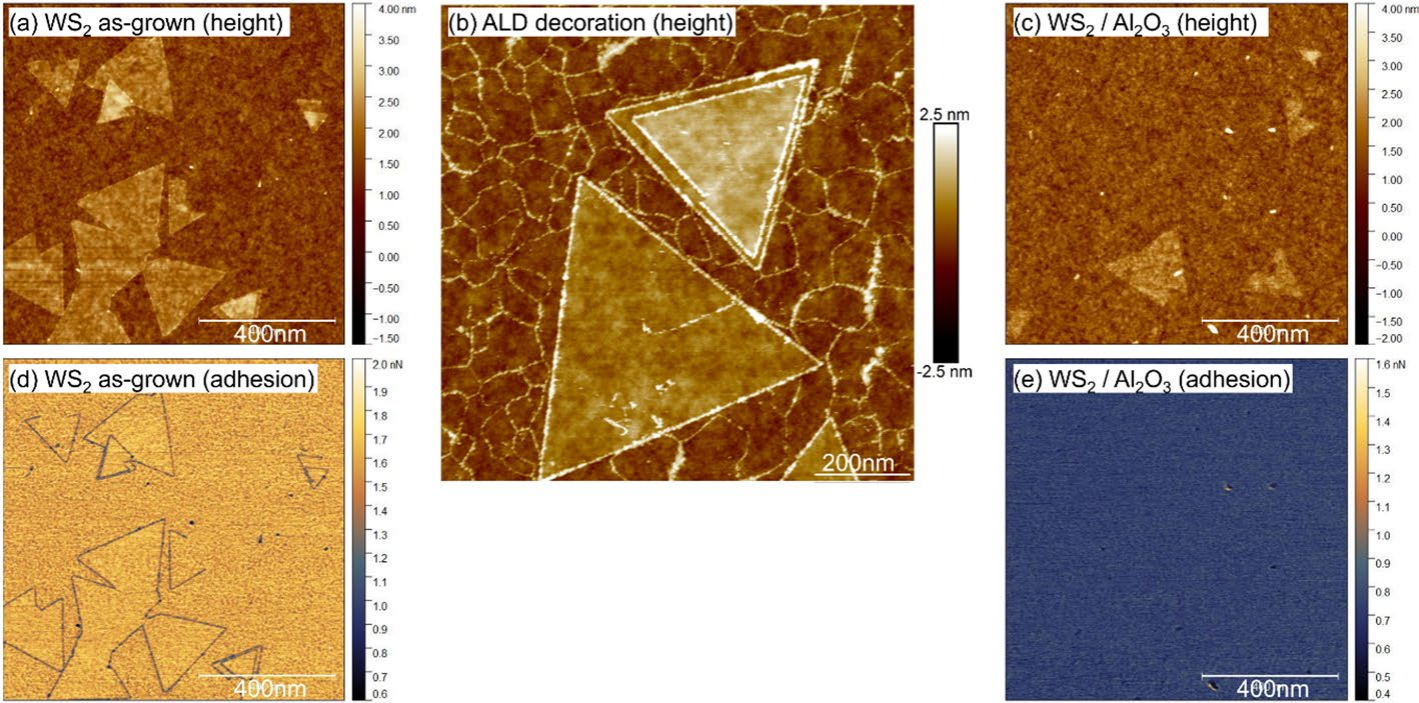
A comparison of (**a**) as-grown WS_2_ and (**b**) after Al_2_O_3_ ALD deposition, which only decorates the defects and grain edges after 10 ALD cycles of TMA/H_2_O. (**c**) After multiple static exposures to TMA at 50 °C, an Al_2_O_3_ thickness of approximately 1.5 nm is achieved with low rms roughness 0.27 nm. RMS values are extracted from the ML region, and the Al_2_O_3_ dep. causes an increase in roughness of only < 0.1 nm. (**d**-**e**) Adhesion channel image measured from the force required to retract the AFM tip from the sample [26], corresponding to the height profile images of (**a**) and (**c**) respectively. (**d**) The grain boundaries of the secondary islands are visible due to edge oxidation evidence of the contrast between WO_x_ and WS_2_. (**e**) A lack of any adhesion features indicates Al_2_O_3_ film closure, as any voids or pinholes present where WS_2_ is exposed would result in a material contrast

**Fig. 11:**
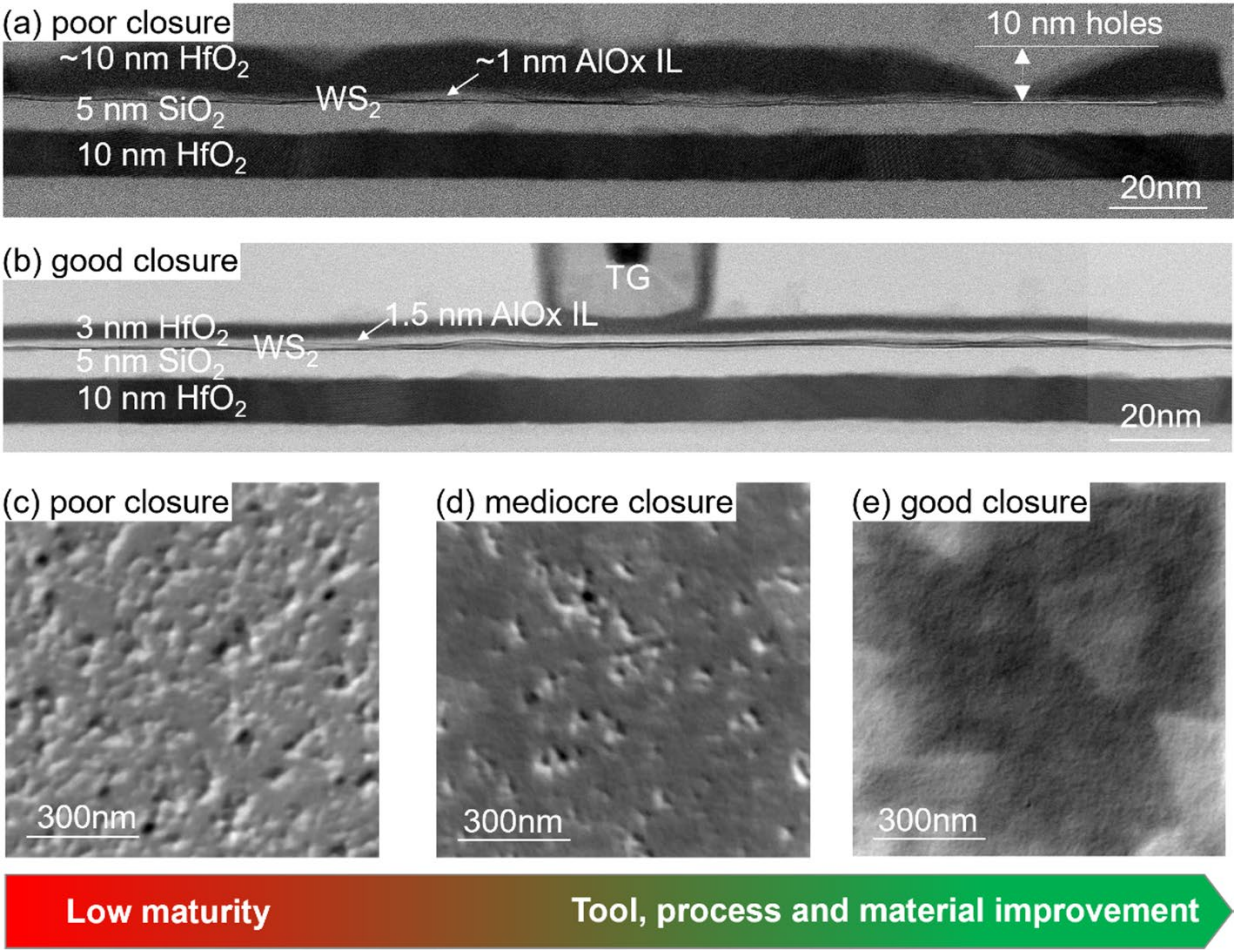
(**a**) TEM of WS_2_ channel with Al_2_O_3_ interlayer with poor uniformity resulting in bad HfO_2_ closure with 10 nm pit-holes. (**b**) TEM of WS_2_ channel and highly uniform Al_2_O_3_ interlayer by optimized reactor conditions (TMA soak at 50 °C), enabling the thinner and uniform 3 nm HfO_2_ cap layer. (**c**-**e**) Top-view SEM images of WS_2_/Al_2_O_3_/5 nm HfO_2_ with poor, mediocre and good closure, respectively

Growing ultrathin uniform dielectrics on 2D materials by ALD methods is challenging, owing to a lack of surface sites required to initiate film nucleation. To initiate the nucleation, we developed a technique [26] to adsorb Tri methyl Aluminum (TMA) – a commonly used precursor for ALD growth of Al_2_O_3_ - uniformly on the TMDC over the complete surface area of the 300 mm wafer. TMA has a relatively high vapor pressure (27 Torr at 40 °C), which allows it to be vapor drawn in high concentrations into an evacuated reactor without the need for an inert carrier gas flow. The reactor is then sealed, allowing TMA adsorption. After an extended period of static exposure at 50 °C, the TMA is removed from the chamber, and a H_2_O pulse affixes the adsorbed precursor network to the surface while removing -CH_3_ ligands. This process is repeated several times until film closure is achieved as shown in Fig. 10(c). The TMA adsorption is significantly higher compared to a pulse/purge scheme in conventional ALD which mostly decorates defects, as highlighted in Fig. 10(a, b). Furthermore, Al_2_O_3_ film closure is evidenced by a lack of features in the adhesion map (Fig. 10(d, e)). The Al_2_O_3_ thickness is typically close to 1.5 nm as shown by cross-sectional TEM in Fig. 11(b). The thickness uniformity was confirmed by comparison of TEM imaging at 10 and 130 mm from the wafer center.

The next step is ALD of the HfO_2_ cap, for which the Al_2_O_3_ interlayer serves as the seed layer. HfO_2_ is grown by ALD form HfCl_4_/H_2_O at 300 °C. HfO_2_ is selected as the capping for the following reasons: (1) it protects the TMDC from plasma damage during active patterning and from ambient conditions. (2) It has a high dielectric constant which is beneficial for a scaled top effective oxide thickness (EOT). (3) It serves as an etch stop layer during ILD0 trench etch for the M0 module (Sect. 2.9) and TG module (Sect. 2.10).

During early iterations, the Al_2_O_3_ interlayer and HfO_2_ cap had poor uniformity due to low tool and process maturity, resulting in 10 nm pit-holes as shown by Figs. 3 and 11(a, c). Through extensive tool and process optimization, a very uniform 1.5 nm Al_2_O_3_ interlayer is demonstrated in Fig. 11(b), enabling the nucleation of a thinner 3 nm cap layer. Top-view SEM images in Fig. 11(e) of the optimized interlayer process with 5 nm HfO_2_ show the absence of pit-holes, and the WS_2_ secondary layer islands can be distinguished through the HfO_2_.

### Active patterning

During active patterning, the TMDC channel, Al_2_O_3_ interlayer, and HfO_2_ cap are patterned by lithography and etching for two reasons: (1) to define the length and width of the test structures and (2) to mitigate delamination of the van der Waals-bonded channel caused by shear forces in subsequent process steps. Patterning provides additional anchoring with the surrounding dielectric, which helps redistribute stress. The shear force acting on the channel is proportional to its area, dictating the delamination sensitivity. Consequently, a wafer fully covered with an unpatterned TMDC is most prone to delamination during later processing. For this reason, the active pattering is performed as early as possible in the process flow, although the step itself remains prone to delamination-induced failure.

**Fig. 12:**
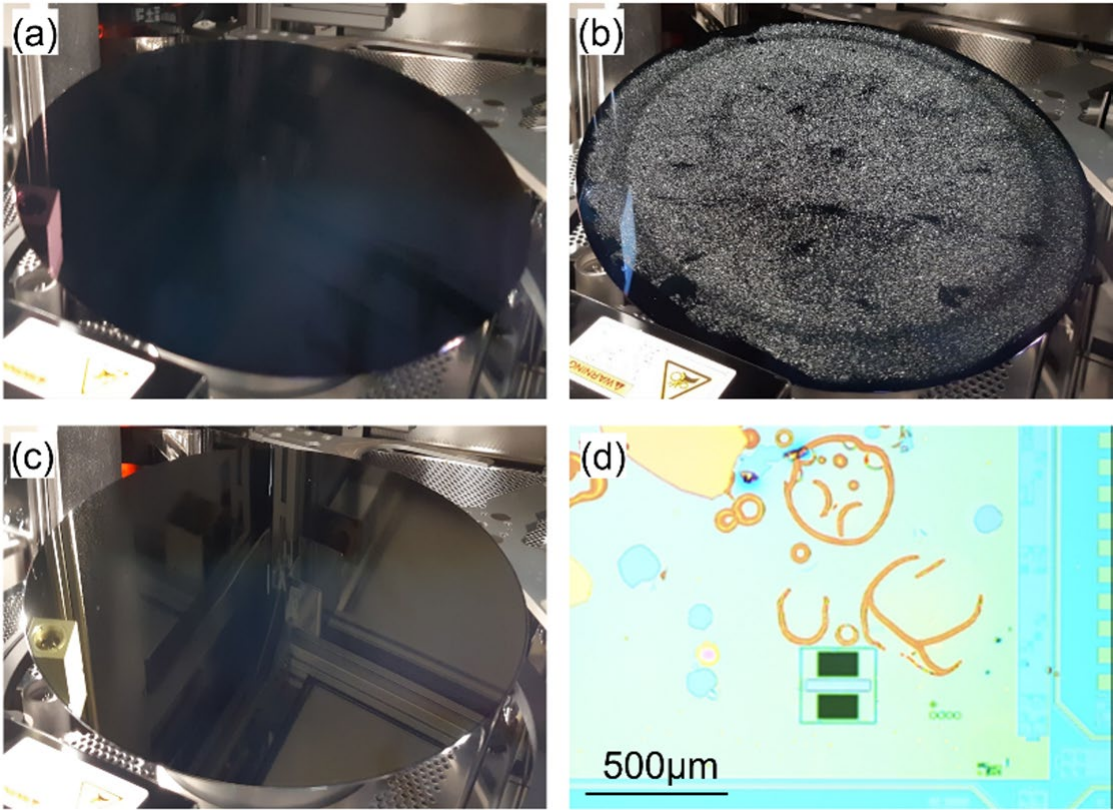
(**a**) 300 mm wafer after WS_2_ channel, interlayer and HfO_2_ cap deposition. (**b**) Severe delamination occurs after SiO_2_ hard mask deposition. (**c**) No delamination occurs with SoC/SoG soft mask deposition. (**d**) Large active areas buckle and delaminate during later processing

To minimize such failures, the mask material used for active patterning must exhibit low internal stress. Stress can arise from intrinsic film stress, or from thermal stress related to the thermal expansion coefficients of the materials and the deposition temperature. While a wafer with WS_2_ capped with Al_2_O_3_/HfO_2_ looks pristine (Fig. 12(a)), deposition of a SiO_2_ hard mask (HM) causes severe delamination (Fig. 12(b)). This delamination can be avoided by using a low-stress and low-temperature soft mask stack consisting of Spin-on Carbon (SoC)/Spin-on Glass (SOG)(Fig. 12(c)). While the SoC/SoG stack is conventionally used to pattern a hard mask, in this work it is utilized directly as the mask itself. The detailed process steps for active patterning are shown in Supplementary information Figure S2. The active stack consisting of the channel, interlayer and cap is etched in a single step due to the lack of etch selectivity between the HfO_2_ cap and the monolayer TMDC. After the active etch, the SoC removed by oxygen plasma stripping, during which the HfO_2_ cap protects the TMDC channel from plasma damage, thereby demonstrating the advantage of the capping-first approach.

**Fig. 13:**
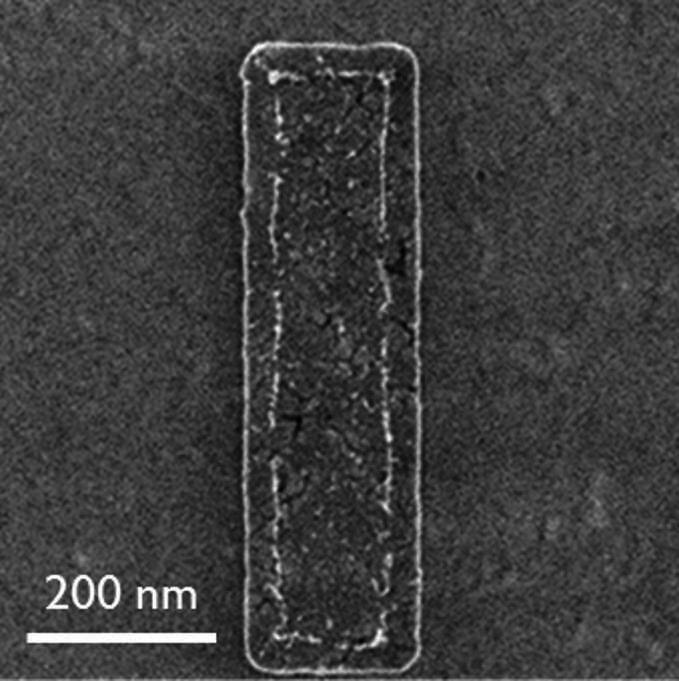
After active patterning and mask strip, hardened mask residues are visible close to the active area edge

After the oxygen plasma-based mask strip, a hardened residue is observed near the active edges, as shown in Fig. 13(a). This residue could, in principle, be removed by a wet strip following the dry etching. However, wet processing steps are avoided when the active edges are exposed, as TMDC layers are highly susceptible to intercalation-assisted delamination. The residue therefore remains on the HfO_2_ cap. The influence of the residue can be avoided in the critical device areas with proper test structure design. Moreover, the residue does not pose issues for the contact and top gate formation, as it is removed during the trench etches.

Observations during later processing confirm that the delamination probability is proportional to the active area [8]. Areas ≥ 100 × 100 µm^2^ readily delaminate, as shown by the stress-induced buckling in Fig. 12(d), while areas ≤ 10 × 10 µm^2^ are preserved.

### M0 contact module

**Fig. 14:**
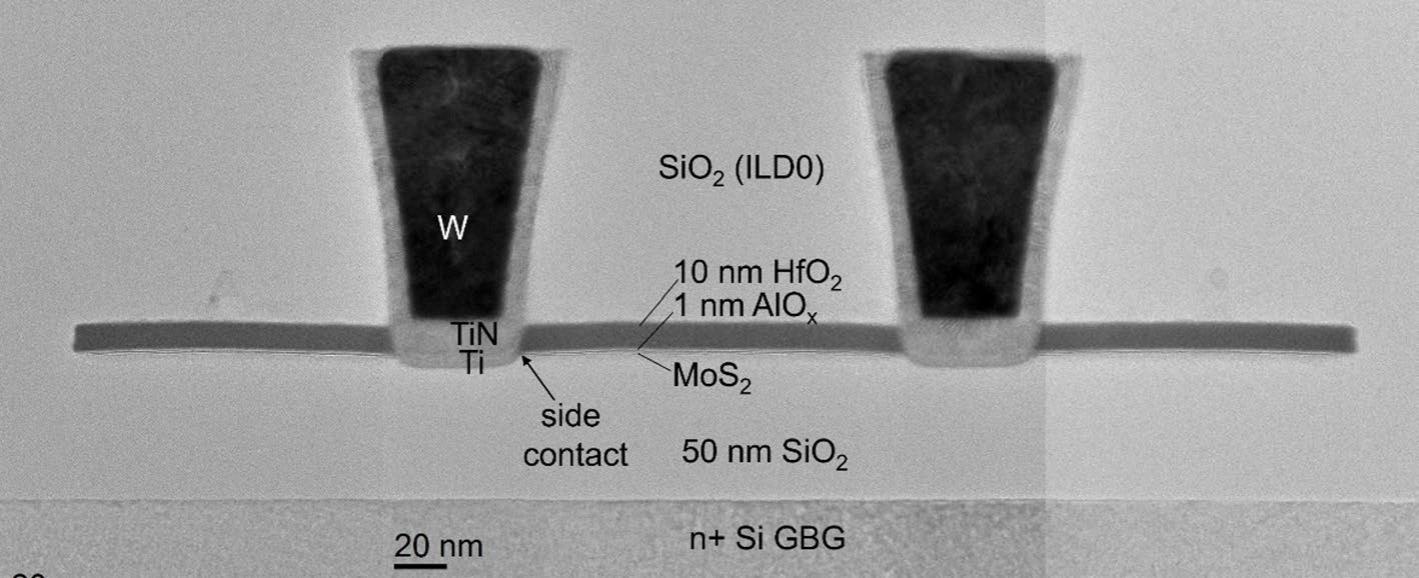
TEM showing the Ti/TiN/W M0 contacts in place. The device has a global back gate configuration with 50 nm SiO_2_ as back gate dielectric and a transferred MoS_2_ channel

The M0 contact module is inspired by the conventional M0 damascene contacts used in Si-channel FETs. In the case of our 2D FETs, the M0 metal plugs serve as the source and drain electrodes, forming a direct metal-semiconductor contact. No intentional doping is applied to the TMDC. Instead, the device operation relies entirely on electrostatic doping induced by the back gate. A representative TEM image of the finalized M0 module is shown in Fig. 14, illustrating the side contact configuration, where the Ti metal makes contact with the edge of the TMDC channel. The illustrated fabrication sequence of the M0 contact module is provided in Supplementary information Figure S3, and a description is given below.

The first process step of the M0 contact formation involves depositing SiO_2_ to encapsulate the patterned active regions. The SiO_2_ layer is then planarized by CMP to improve the lithographic resolution, and to facilitate the metal CMP at the end of the module. After planarization, the resulting ~ 100 nm SiO_2_ layer is known as the interlayer dielectric (ILD0), which fully encapsulates and protects the TMDC. Subsequently, M0 trenches are defined using a 2-step etch process. In the first step, the trenches are etched into the SiO_2_, stopping on the HfO_2_ cap, which acts as an etch stop, followed by an oxygen plasma-based mask strip. In the second step, the etch continues through the HfO_2_ cap, the interlayer, through the TMDC channel and into part of the back gate dielectric. The lack of dry etch selectivity between HfO_2_, Al_2_O_3_, and the monolayer TMDC implies all materials are etched in a single step. Consequently, the side contact configuration imposes itself and the etch duration is extended to ensure an appropriate trench angle that exposes the TMDC edge.

In the global back gate configuration, there is a risk that the M0 etch penetrates too deeply into the back gate dielectric which can lead to dielectric breakdown during device operation, as illustrated in Fig. 4(b). In contrast, Fig. 14 shows an example where the M0 etch is within specifications. To prevent severe over-etching, the M0 design (which will be discussed later in Sect. 3.1 and Fig. 17(a)) is such that the contact trenches are fully enclosed within the active region, such that the HfO_2_ cap within the active can function as an etch stop layer during the first part of the M0 etch. A more robust M0 etch process is achieved with the triple layer global back gate dielectric stack consisting of 50 nm SiO_2_/10 nm HfO_2_/5 nm SiO_2_, where the HfO_2_ acts as an additional etch stop layer for the second part of the M0 etch, as discussed in Sect. 2.4.

After etching, the M0 trenches are filled with a triple layer metal stack. The contact metal is Ti, deposited by physical vapor deposition (PVD). Subsequently a TiN liner is deposited by ALD to serve as an oxidation barrier for Ti, and to enhance the adhesion of the fill metal. The fill metal is tungsten (W), deposited by a combination of ALD and chemical vapor deposition (CVD). Finally, the metal stack is planarized by CMP, completing the M0 module as shown in Fig. 14.

### Top gate module

**Fig. 15:**
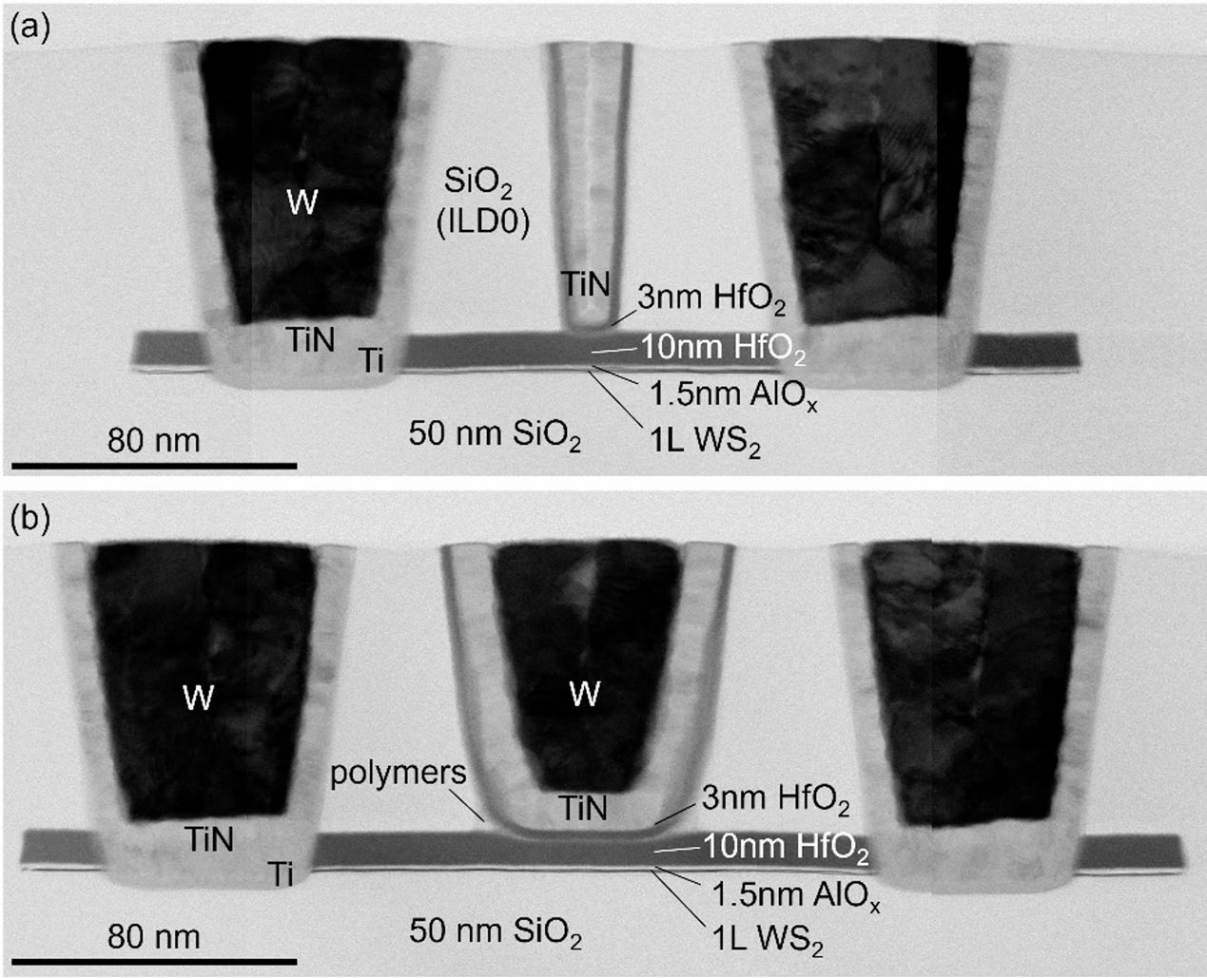
TEM of WS_2_ channel device after top gate fabrication with (**a**) L_ch_=70 nm and L_tg_=40 nm (mask values) and L_ch_=107 nm and L_tg_=11 nm (TEM-calibrated). (**b**) A slightly longer device with L_ch_=130 nm and L_tg_=100 nm (mask values) and L_ch_=159 nm and L_tg_=43 nm (TEM-calibrated). The discrepancy between mask values and TEM-calibrated values arises due to the tapering of the trenches. Polymeric residues are seen at the edges of the top gate trench

In the top gate module, the top gate electrodes are also fabricated using a damascene-type process. The design, shown in Fig. 1 and later discussed in Sect. 3.1 and Fig. 17(e), positions the top gate line between the M0 trenches with a 15 nm margin, and extends it longitudinally across the active region. This way, the entire channel width is electrostatically controlled, while only part of the channel length is gated, as shown by the TEM in Fig. 15. The implications of this design on the device operation will be discussed in Sect. 3.1.

The detailed process steps for the top gate formation are shown in Figure S4 of the Supplementary Information. The process closely follows that of the M0 contact formation, with several modifications. First, a 5 to 10 nm SiO_2_ layer is deposited to cover the M0 contacts, enabling lithographic rework without damaging the contacts. The TG trench etch is identical to the M0 SiO_2_ trench etch, which stops on the HfO_2_ cap. The top gate fill consists of three layers. First, 3 nm HfO_2_ is deposited by ALD to avoid a short circuit between the subsequent gate electrode and the exposed TMDC channel at the edges of the active region where the top gate trench overlaps. Next, the top gate electrode is formed, consisting of a conformal TiN layer deposited by ALD, which is well suited for filling narrow trenches, followed by a W fill deposited sequentially by ALD and CVD. The top gate module is completed with a planarization CMP.

The final structures are shown in Fig. 15(a-b). In the top gate region, the interface between the HfO_2_ cap and the 3 nm HfO_2_ top-up layer indicates the 2 nm recess into the HfO_2_ cap. This suggests that some plasma-induced damage may occur in the dielectric ~ 8 nm from the TMDC channel. Furthermore, there is a discrepancy between the designed top gate dimensions and effective dimensions observed in the TEM images in Fig. 15(a-b). Nominal top gate lengths of 40 nm and 100 nm on the mask correspond to effective lengths of 11 nm and 43 nm, respectively. These reduced dimensions result from both trench tapering and etch loading effects in narrow trenches, which cause variations in trench width and depth. Additionally, the etch chemistry forms polymer residues, visible in Fig. 3(a) and Fig. 15(b). This reduces the reliability of the narrow trenches. Therefore, the focus is placed on mask designs with L_tg_≥100 nm (corresponding to 43 nm effective length). For those devices, any remaining polymer residues at the interface between the HfO_2_ cap and HfO_2_ top-up layers could adversely affect the gate stack reliability, evaluated in Sect. 3.6, but its impact has not been quantified yet.

After gate metal CMP, occasional gate rip-out is observed when the gate-to-active overlap area exceeds approximately 4 × 4 µm^2^, as illustrated in Supplementary Information Figure S5. This gate rip out is attributed to the large top gate electrode area combined with the weak van der Waals interface of the underlying TMDC and the considerable shear forces generated by CMP. Therefore, the highest yield is achieved for mask designs 100 nm ≤ L_tg_ ≤ 1 μm. The yield is further discussed in Sect. 3.3.

### Via to bottom module

The Via To Bottom (VIATB) provides an electrical connection between a top-side probe pad and the BGC, as illustrated in Fig. 2(b). In the global back gate configuration, the VIATB is optional, since the wafer backside can alternatively serve to make electrical contact. The fabrication steps for the VIATB are detailed in Supplementary Information Figure S6. The VIATB dimensions are on the order of 100 nm, as aggressive scaling is unnecessary for these semi-isolated devices.

The etching and metallization processes used for VIATB follow conventional Si-based process technology. Since the VIATB is placed outside the active region, it is not affected by the presence of the TMDC. Conventional oxide etches stopping on Si are employed. The metallization stack consists of Ti/TiN with a W fill, which is commonly used for contacts to the Si bulk.

### VIA and M1 modules

**Fig. 16:**
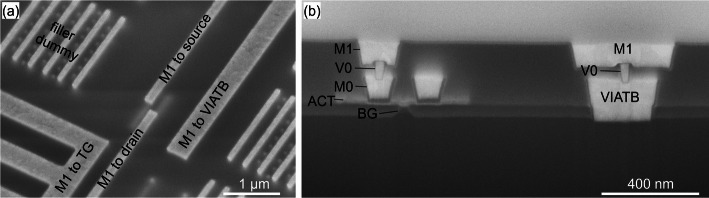
(**a**) Tilted SEM taken after M1 processing. (**b**) SEM of a fully processed device with a local TiN back gate and transferred WS_2_, and FIB cross section

The VIA and M1 modules are implemented using a conventional W-based middle of line process, where the VIAs and M1 are integrated one after the other. The use of W allows for higher thermal budget in post processing anneal testing. The related process steps are shown in Supplementary Information, Figure S7. In the fabricated devices, the VIAs have a size of 45 nm, and the M1 lines are 105 nm wide. For devices with short channel length (M0 spacing) the M1 lines arrive on both sides of the device as is the case for the example in Fig. 16(a). This is required to respect the minimal M1 to M1 spacing design rule. A focused ion beam (FIB) cut of a fully processed device is shown in Fig. 16(b).

## Device performance and yield

### FET operation with different gate configurations

**Fig. 17:**
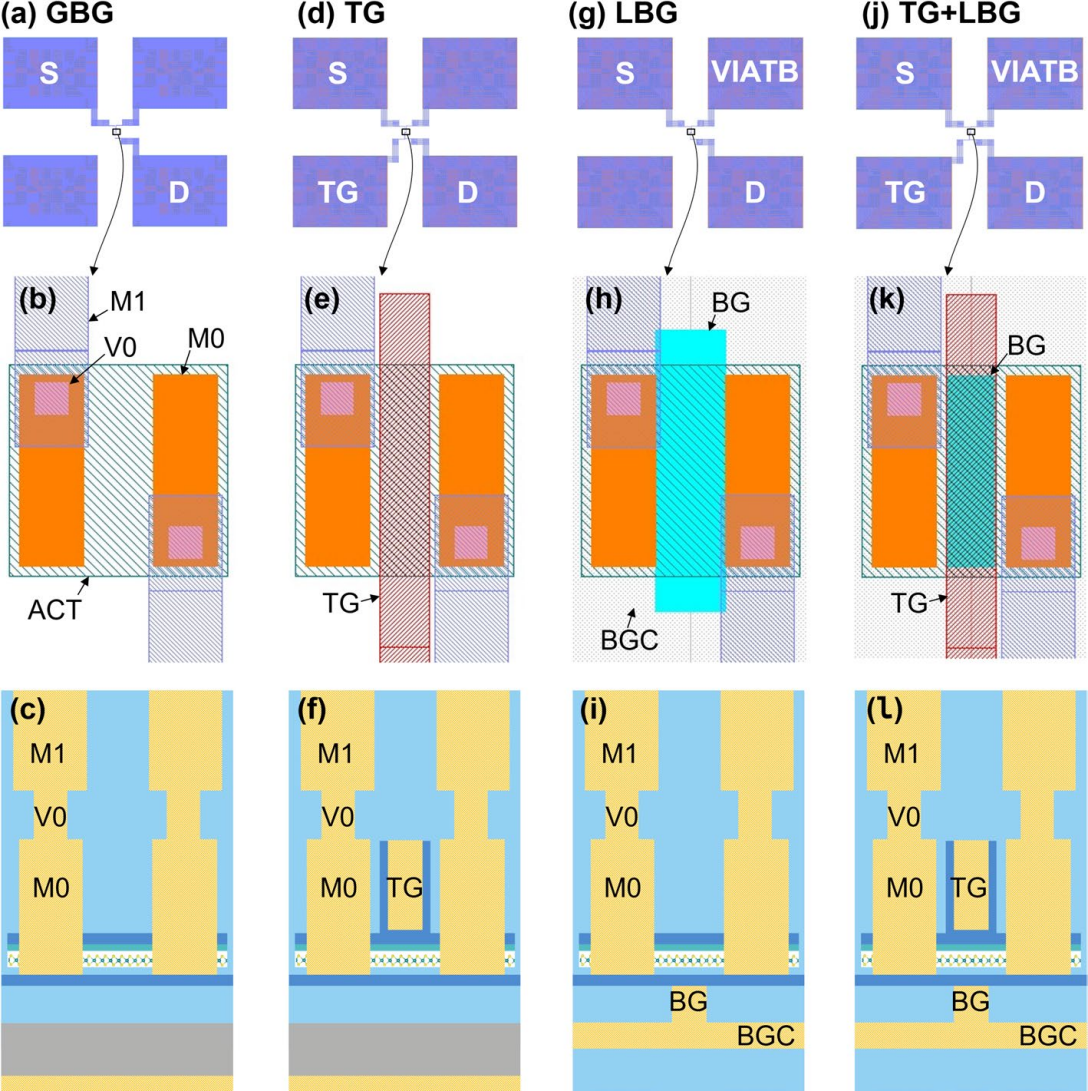
The four types of gate configurations are (**a**-**c**) global back gate, (**d**-**f**) top gate, (**g**-**i**) local back gate and (**j**-**l**) top gate + local back gate.

This section covers the operation of FETs with the four previously discussed gate configurations: global back gate (GBG), top gate (TG), local back gate (LBG) and top gate + local back gate (TG + LBG). The four device types are shown in Fig. 17. The benefits and drawbacks of each gate configuration are shown in Table 2, and are covered in more detail further below. The discussion is focused on the baseline flow with WS_2_ channels by monolithic deposition.

**Table 2:** Benefits and drawbacks of the different gate configurations.

Configuration	Main benefit & use case	Performance drawback
Global back gate (GBG)	Channel mobility and contact resistance extraction	Back EOT scaling not possible due to side contacts damaging the back gate dielectric
Top gate (TG)	Top oxide uniformity and scaling studies	Series resistance due to ungated contacts and access regions
Local back gate (LBG)	Highly scaled back gate EOT when combined with transfer to high-k dielectric	Series resistance due to ungated contacts and access regions, LBG topography
Top gate + local back gate (TG+LBG)	Most scaled dual gate EOT for steepest subthreshold swing (SS)	Series resistance due to ungated contacts and access regions, LBG topography

**Fig. 18:**
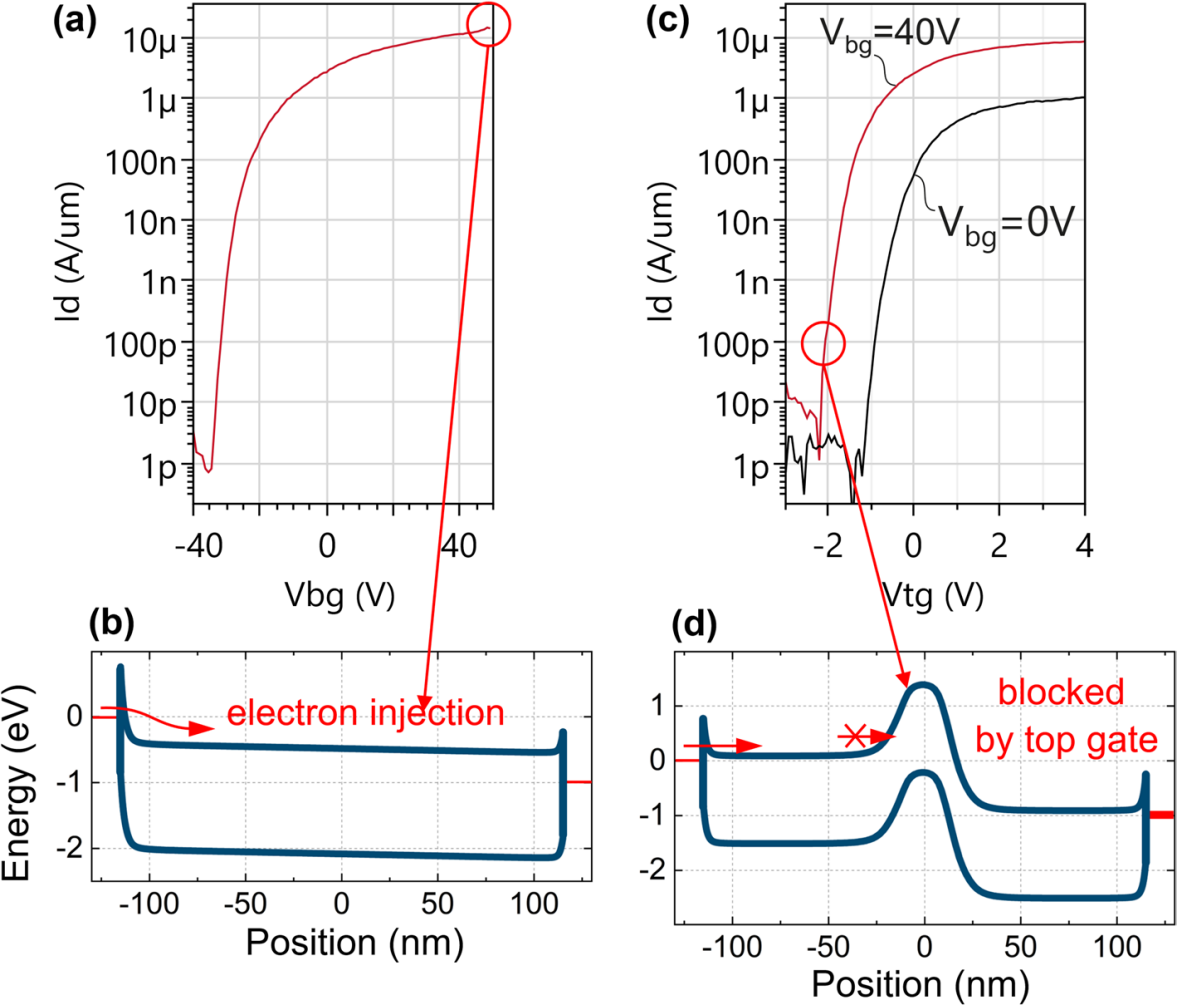
(**a**) GBG device operation with back gate sweeps. V_d_=1 V, L_ch_=559 nm and W_ch_=500 nm. (**b**) Band diagram of GBG device at high V_bg_ (on-state). (**c**) TG device transfer characteristics with top gate sweep and back gate stepping. L_tg_=433 nm, L_ch_=559 nm, W_ch_=500 nm. (**d**) Band diagram of TG device at high V_bg_ but low V_tg_, blocking the electron flow in the middle region of the channel. Band diagrams are generated with Sentaurus Device [28]

The design of the global back gate configuration is shown in Fig. 17(a-c). It is the simplest configuration and has a thick back gate EOT of either 50–57 nm (stacks in Sect. 2.4). Transfer characteristics of GBG FETs with WS_2_ channel are shown in Fig. 18(a). The back gate voltage is applied to the backside of the wafer via the chuck of the measurement tool. As the back gate voltage is swept from − 40 to 50 V, the channel accumulates electrons and the transistor turns on. The Ti side contacts are not optimized and form high-resistance Schottky barriers with the WS_2_. As the global back gate is swept to higher voltages, the contact regions are electrostatically n-doped and the contact resistances are lowered. The band diagram in Fig. 18(b) shows the electron current path in the on-state with tunnelling through the reverse biased source Schottky barrier, drift along the channel and tunnelling through the forward biased drain Schottky barrier. The transfer characteristics show dominant n-type conduction, likely caused by traps with energetic alignment with the lower half of the WS_2_ bandgap. These prevent Fermi level movement to the valence band edge and prevent hole accumulation [27].

The global back gated device is best suited for contact resistance extraction and mobility extraction. It is the configuration of choice to develop and improve the TMDC deposition and transfer processes. The drawback of this configuration is that the side contacts prevent back EOT scaling. Dielectric breakdown would readily occur in the oxide regions directly below the M0 trenches as it is weakened by plasma damage, as shown in Sect. 2.4 and Fig. 4(a).

The transfer characteristics of top gated (TG) devices are shown in Fig. 18(c) and can be understood as follows. The design in Fig. 17(d-f) and the TEM in Fig. 15(b) show the middle portion of the channel is controlled by both the top gate (EOT = 3 nm) and global back gate (EOT = 50–57 nm). The rest of the active area is electrostatically controlled by the global back gate. This critically includes the Schottky contacts and the access regions, which are the portions of channel to the left and right of the top gate. The access regions are 45 nm to 60 nm long due to the re-entrant slopes of the M0 and top gate trenches (Fig. 15(b)). These slopes systematically limit access region length scaling which induces significant resistance. The transfer characteristics in Fig. 18(c) show that when both gates are positively biased (V_tg_=4 V, V_bg_=40 V), the on-current is nearly identical to the one of the global back gated device in Fig. 18(a). The band diagram in Fig. 18(d) shows that when the top gate is set to a low voltage, the electron flow is blocked by the energy barrier in the middle of the device. Figure 18(c) also shows that lowering the global back gate to 0 V causes a positive V_t_ shift of the top gate transfer characteristics, because it lowers the energy barrier in the middle portion of the channel. Additionally, lowering the global back gate to 0 V also causes a drop in on-current due to higher contact resistances and access resistances. A breakdown of resistances by the top-gated channel region, access regions and contact regions is performed in [12]. The top gated configuration is best suited for top EOT uniformity and scaling [29] and defectivity studies [30]. Comparisons of the on-current of GBG-controlled devices and TG-controlled devices should be treated with utmost care, because only the GBG has electrostatic control over the entire channel length, including the contact interfaces, and can therefore enable a lower resistive path.

The design of local back gated (LBG) devices is shown in Fig. 17(g-i), and also features ungated access regions caused by the re-entrant M0 slopes. The device operation is similar to top gated devices, but without the separate control by the global back gate regions over the access regions. Therefore, the local back gated devices are also strongly affected by series resistance, with an on-current capped around 1–10 µA/µm depending on the uncontrolled doping of the access regions. Example transfer characteristics with doped Silicon local back gate and TiN local back gates will be discussed later in Sect. 3.3.3 and Fig. 23.

The design of top + local back gated (TG + LBG) devices is shown in Fig. 17(j-l). These have the benefit of combined scaled EOT for better electrostatic control (steeper SS) at short gate lengths, but they also suffer from series resistance like in the previous cases. This has been described in [12] along with a Technology Computer-Aided Design (TCAD) performance projection.

### Four-point probe mobility and contact resistance

Channel mobility and contact resistance are two critical parameters influencing the FET on-current, a key performance metric for any transistor. Despite considerable efforts to optimize mobility and contact resistance in FAB 2D devices, improving either of them remains a challenging task. The baseline WS_2_ channel by monolithic deposition yields small grains (a few tens nm) and numerous grain boundaries (Sect. 2.5.1). This high defectivity leads to a low mobility of ~ 3 cm^2^/V∙s [12, 16, 17]. Regarding the contact resistance, the best performance has been demonstrated with Bi, Sb, Y in the top contact configuration [4–6]. In our baseline flow, the channel is immediately capped by a dielectric, which makes landing on atomic-thin 2D channels during the contact etch step very challenging. Consequently, side contacts are employed (Sect. 2.9), though these are commonly associated with high contact resistance [8, 9, 12, 16, 18].

Since improving mobility and contact resistance require different device adjustments, it is important to accurately extract these parameters during development and to avoid their convolution. Field effect mobility is derived from the transistor transfer curve; however, the high contact resistance typical for FAB 2D devices can cause mobility underestimation - even in long channel devices. Conversely, contact resistance is usually extracted from the infinite overdrive method or transfer length method (TLM) [31], which rely on extrapolation and assume equal drain and source contact resistance R_S_ and R_D_, leading to certain estimation errors. Additionally, the WS_2_ FETs have high defectivity which prevents identification of the g_m_ peak and the threshold voltage, which prevents the use of the TLM. This will be further discussed in Sect. 3.3.1 and Fig. 25(b).

**Fig. 19:**
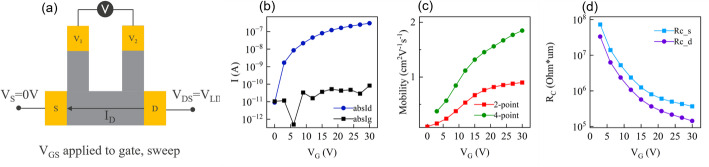
(**a**) A schematic illustration of the four-point test structure from which we obtain (**b**) the transfer characteristics, (**c**) the two- and four-point field-effect mobility, (**d**) the source and drain contact resistance. The test structure has a WS_2_ channel and Ti side contacts. resistance

To address these issues, four-point probe test structures with global back gate configuration are employed to separate the mobility and contact resistances. The approach is illustrated in Fig. 19(a). The source, drain, and global back gate of the test structure are biased to measure transfer characteristics in Fig. 19(b), while two Kelvin probes along the channel measure the voltage drop V_12_ between them. Knowing V_12_ and I_D_ allows calculation of the voltage drops at the source and drain contacts through extrapolation of the linear electrostatic potential profile. Furthermore, V_12_ can substitute V_SD_ in the field effect mobility calculation ($\:{{\upmu\:}}_{\mathrm{F}\mathrm{E},4\mathrm{P}\mathrm{P}}=\frac{{\mathrm{g}\mathrm{m}}_{\mathrm{M}\mathrm{A}\mathrm{X}}\mathrm{*}{L}_{\mathrm{C}\mathrm{H}}}{{W}_{\mathrm{C}\mathrm{H}}\mathrm{*}{C}_{\mathrm{O}\mathrm{X}}\mathrm{*}{V}_{12}}$), eliminating the impact of contact resistance.

As a result, four-point measurements allow to deconvolute mobility, R_S_, and R_D_ and extract them more accurately than using two-point measurements. As illustrated in Fig. 19(d), the contact resistances of a transistor with baseline WS_2_ channel and Ti side contacts are gate bias dependent and on the order of several 10–100 kΩ∙µm. Separating the contact resistances and channel sheet resistance produces a two times higher mobility than for two-point measurements in Fig. 19(c). This effect will be further discussed in Sect. 3.4.1 and Fig. 26, where the four-point mobility over several wafers and lots with WS_2_ channel is systematically higher than the two-point mobility. The field-effect mobility of MoS_2_ will be further evaluated in Sect. 3.3.2.

The high contact resistances could have several causes. Firstly, during the M0 trench etch, the exposed edges of MoS_2_ and WS_2_ could be affected by plasma damage. Secondly, the exposed monolayer edge could be affected by the air break between etch and metallization, or by the high-energy Ti metal sputtering. Finally, Ti is known to cause Fermi level pinning, even for evaporated top contacts [5, 32]. The mobility of the WS_2_ and MoS_2_ channels and their Ti side contact resistances are summarized in the Supplementary Information table S1.

### Yield evaluation

#### Yield for global back gated devices (monolithic WS_2_)

**Fig. 20:**
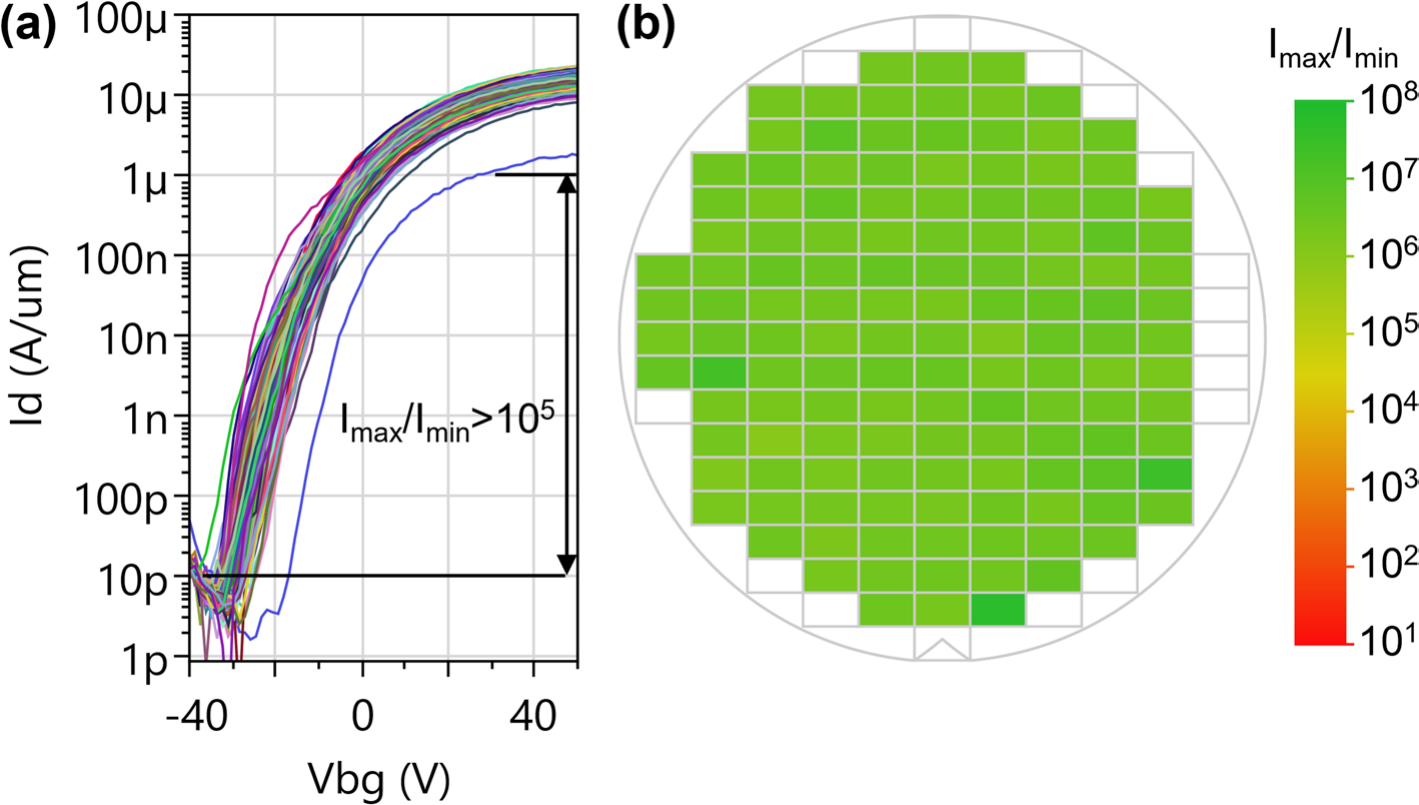
(**a**) Transfer characteristics of FETs with monolithic WS_2_, global back gate configuration, L_ch_=194 nm, W_ch_=1 μm, V_d_=1 V. The yield criterion I_max_/I_min_>10^5^ is reached for all measured devices (133/133). (**b**) Wafer map of the same devices showing I_max_/I_min_ ratio across the wafer

We define the yield for global back gated devices with monolithic WS_2_ as the percentage of devices with channel dimensions L_ch_~200 nm, W_ch_=1 μm that reach I_max_/I_min_>10^5^ using a gate sweep range within the breakdown limits and at V_d_=1 V. The analysis excludes devices in the outermost 1.5 cm of the wafer (edge exclusion zone). Figure 20 shows the yield is 133/133 (all measured devices are operational) hence > 99%. Global back gate devices with monolithic WS_2_ have the simplest configuration and least processing steps, hence highest yield.

Table 3 shows that as the process complexity increases (transferred channel instead of monolithic, addition of a local back gate, a top gate) the yield suffers penalties. A drop in yield would be observed if the following occurs:

- The channel has incomplete coverage resulting in open circuit devices.
- The channel or gate oxides have too high defectivity. The channel is insulating or metallic resulting in poor modulation.
- The channel is strongly n-doped due to deposition or dielectric environment and cannot be depleted within the gate sweep range.
- M0 is not in contact with the channel or there is poor contact resistance.
- CMP causes rip-out of the active stack or ILD0.
- CMP of metal fill is incomplete, causing a short circuit between source and drain.
- The M0 or top gate trench is etched too deep, causing early gate oxide breakdown.

**Table 3:** Yield summary for different gate configurations and channel types

Channel	Gate configuration	Criterion	Yield
WS_2_ direct dep.	Global back gate	I_max_/I_min_ > 10^5^ L_ch_ ~ 200 nm	> 99% (133/133)
WS_2_ direct dep.	Local back gate (Si)	I_max_/I_min_ > 10^5^ L_ch_ ~ 200 nm, L_bg_ = 113 nm	> 95% (18/18)
WS_2_ direct dep.	Local back gate (TiN)	I_max_/I_min_ > 10^5^ L_ch_ ~ 200 nm, L_bg_ = 84 nm	11% (2/18)
WS_2_ direct dep.	Top gate	I_max_/I_min_ > 10^5^ L_ch_ ~ 567 nm, L_tg_ = 434 nm	> 99.8% (695/695)
WS_2_ transferred	Global back gate	I_d_ > 1 µA*W_ch_/L_ch_ L_ch_=194 nm, 1.11 μm, 10.2 μm	95–99%
MoS_2_ transferred	Global back gate	I_d_ > 2 µA*W_ch_/L_ch_ L_ch_=194 nm, 1.15 μm, 10.1 μm	97–99% but strong n-doping

#### Yield for global back gated devices (CoD2W transfer)

**Fig. 21:**
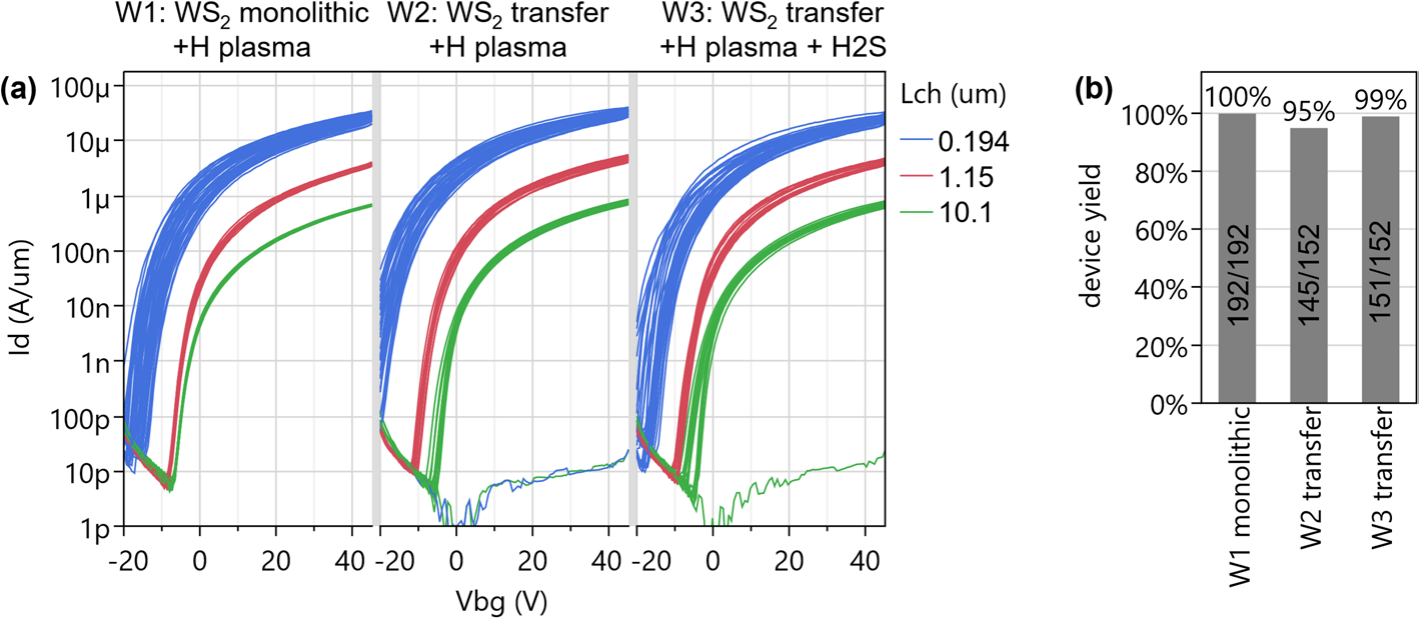
(**a**) The transfer characteristics of wafer 1 with monolithic WS_2_ are nearly identical to W2 and W3 with transferred WS_2_ (same growth recipe, but extra transfer step). (**b**) The yield is 95–99% with rigid carrier transfer process. Reported in [6]

The performance of transferred WS_2_ with a CoD2W process with rigid carrier introduced in Sect. 2.6.2 is evaluated with a global back gated device configuration. Figure 21(a) shows that the transfer characteristics are nearly identical to WS_2_ directly deposited on the device wafer [18], which confirms the damage by the transfer process is negligible compared to defectivity present in the direct-deposited WS_2_. The yield is evaluated differently for these devices, since 2” patches of WS_2_ are transferred to 300 mm wafers and only few devices are available. Therefore, more channel lengths (194 nm, 1.15 μm, 10.1 μm) are considered and the criterion is taken as I_d_ > 1 µA*W_ch_/L_ch_ at V_bg_=20 V and with gate modulation. Figure 21(b) shows the yield for transferred WS_2_ with rigid carrier process is high with 95% for a first wafer and 99% for a second wafer.

**Fig. 22:**
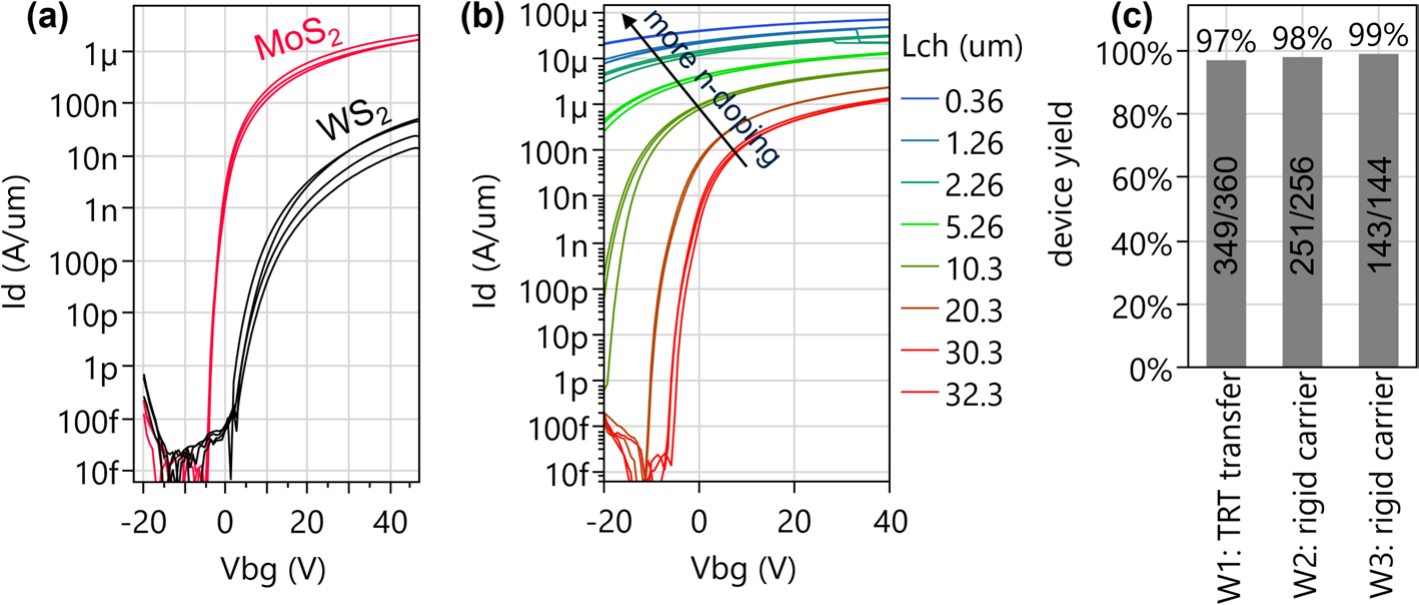
(**a**) Strongly improved long-channel SS and I_on_ for transferred MoS_2_ (templated growth on 2” sapphire) compared to transferred WS_2_ (growth on SiO_2_). L_ch_=30 μm, W_ch_=75 μm, V_d_=1 V, EOT = 50 nm. (**b**) MoS_2_ FETs with shorter L_ch_ have higher on-current but strong negative V_t_ shift by n-doping, preventing off-state read-out. MoS_2_ FETs have W_ch_=75 μm. (**c**) Functional yield of MoS_2_ devices is 98%-99% for rigid carrier transfer (with modified yield definition) as reported in [6]

Patches of MoS_2_ grown by MOCVD on 2” sapphire are also included in the same CoD2W transfer process (optical image shown in Fig. 7(b)). While the WS_2_ is grown on SiO_2_, leading to small defective grains of a few tens nm (Sect. 2.5.1) and a low mobility of ~ 3 cm^2^/V∙s, the MoS_2_ by templated growth on sapphire has much better crystallinity (Sect. 2.5.2), a lower defectivity leading to a higher on-current (I_max_=250 µA/µm for shortest L_ch_) and steeper SS of long channel devices in Fig. 22(a). However, devices with smaller W_ch_ and L_ch_ experience a strong negative V_t_ shift by n-doping of MoS_2_, shifting the off-state out of the measurable range (Fig. 22(b)). This prevents full exploitation of the improved channel quality of MoS_2_. To the best of our knowledge, this effect is unique to our FAB integration process, as lab-based devices made with the same transferred MoS_2_, processed with Ni/Pd liftoff contacts and Al_2_O_3_/HfO_2_ capping are not plagued by this phenomenon. We suspect a chemical doping effect by e.g. hydrogen incorporation, more severely affecting MoS_2_ than WS_2_. Ways to suppress and control it are still in development.

The better crystallinity of MoS_2_ also enables a clear g_m_ peak. This g_m_ peak is absent for WS_2_ and workarounds for benchmarking will be discussed in Sect. 3.4.1. The presence of a gm peak for MoS2 enables the extraction of field-effect mobility and TLM mobility. These are in good agreement with each other, but are also affected by the channel width and the n-doping effect; The mobility is 20–23 cm^2^/V∙s for W_ch_=75 μm and increases to 48–54 cm^2^/V∙s for W_ch_=0.3 μm [18].

The yield criterion for MoS_2_ FETs is defined similarly to that of the transferred WS_2_ FETs; I_d_>2 µA*W_ch_/L_ch_ at V_bg_=20 V and with gate modulation for channel lengths of 194 nm, 1.15 μm, 10.1 μm. The corresponding yields are 98% and 99% for wafers with rigid carrier transfer and 97% for a wafer with thermal release tape (TRT) transfer process (Fig. 22(c)). The key performance metrics for MoS_2_ FETs are summarized in the Supplementary Information table S1.

#### Yield for local back gated devices

**Fig. 23:**
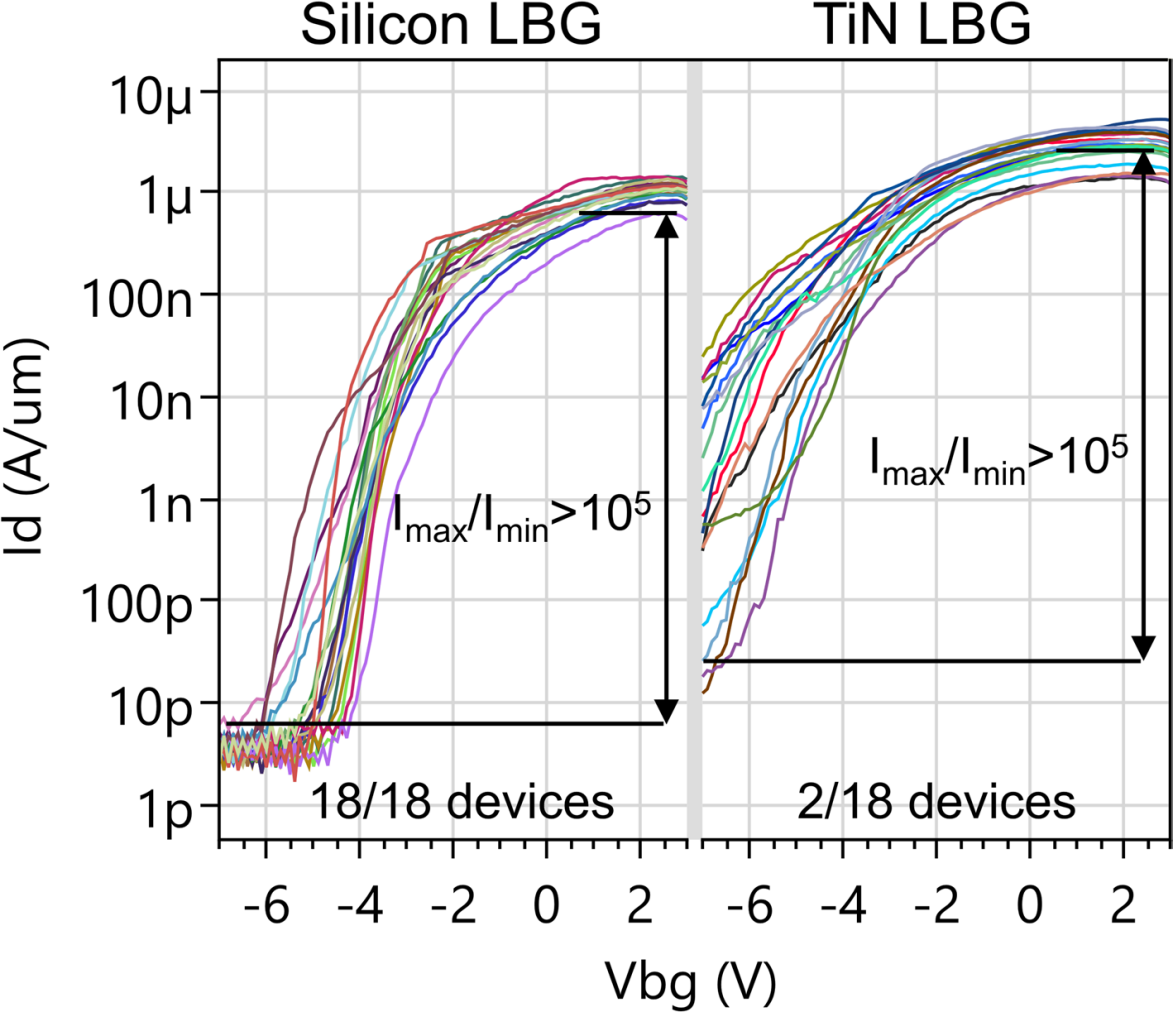
For Silicon local back gates with direct deposited WS_2_, the yield is 18/18 (> 95%). With TiN local back gates the yield is only 2/18 (11%). The calibrated device dimensions are Lch = 189 nm, L_bg_=113 nm for Silicon local back gate, L_bg_=84 nm for TiN local back gate Wch = 1 μm

For local back gated devices (monolithic WS_2_ channel deposition) we use the same yield definition as for global back gated devices. We compare the yield of doped Silicon versus TiN local back gated devices in Fig. 23. The devices are integrated withing BGC, and the local back gate voltage is applied via the backside of the wafer. The local back gate lengths are slightly different for the two materials, as a mask dimension L_bg_=100 nm results in a TEM-calibrated L_bg_=113 nm in the case of Silicon and L_bg_=84 nm in the case of TiN. The channel lengths are longer, L_ch_=189 nm, resulting in incomplete electrostatic control by the local back gate over.

The transfer characteristics of Silicon local back gated devices have kinks, while TiN local back gated devices have a degraded SS (Fig. 23). Both these features are not present in the global back gate characteristics, which is indicative of a less optimal electrostatic control. Possible causes are reduced charge accumulation in the implanted Silicon gate, minor topography (~ 3 nm) for TiN local back gates causing a bending of the channel, and an unintended native oxide at the Si/TiN interface. Figure 23 shows the yield criterion of I_max_/I_min_>10^5^ is met for 18/18 devices in case of Si local back gate devices and only 2/18 for TiN local back gate.

#### Yield for top gated devices

**Fig. 24:**
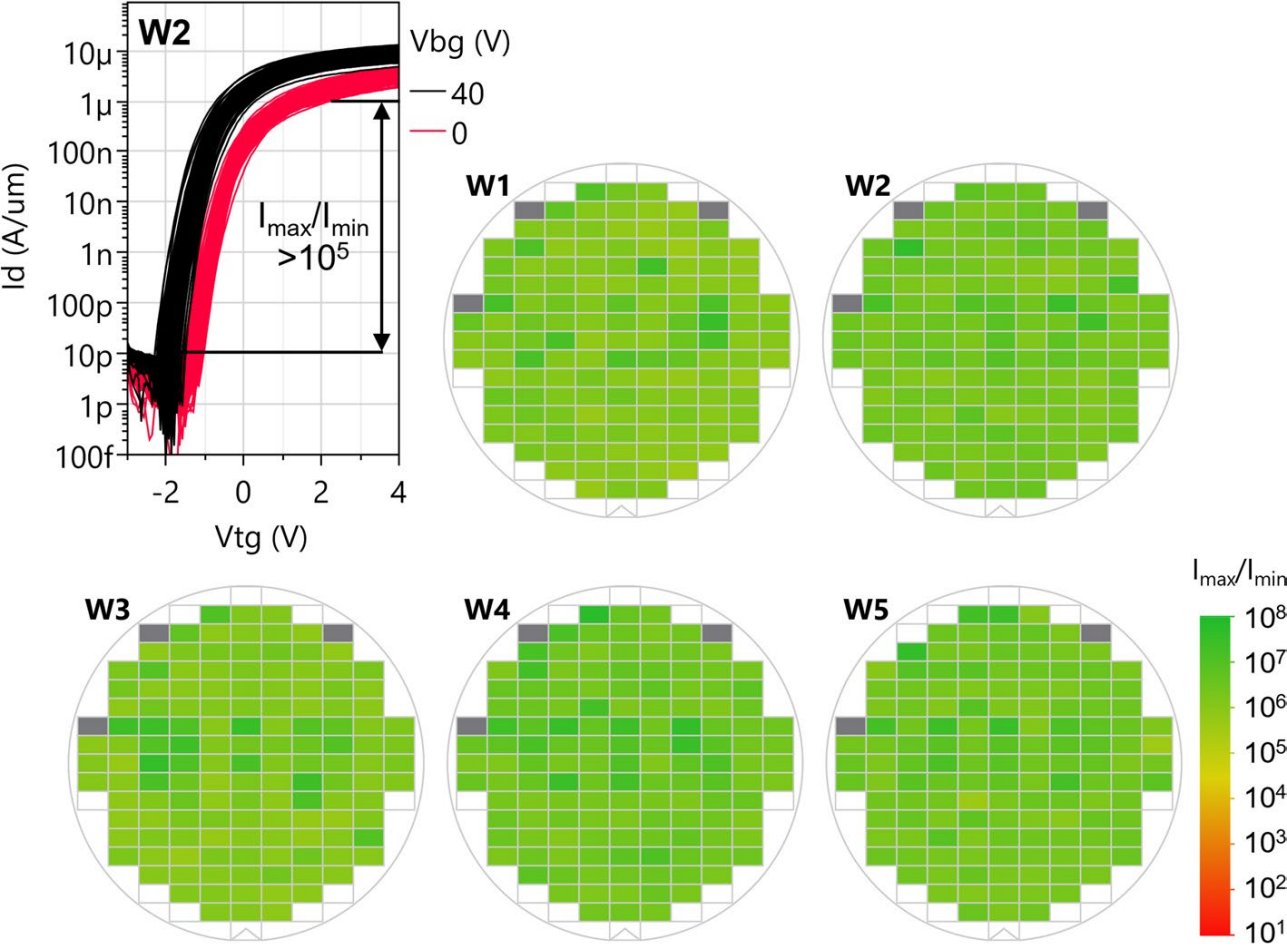
Top gated transfer characteristics of all devices with L_ch_=567 nm, L_tg_=434 nm in wafer 2. The top gated device yield is 99%-100% for the 5 example wafers shown. The edge exclusion zone where the active is removed is not taken into account

For top gated devices with monolithic WS_2_ channel deposition, Fig. 24 shows 116 nominally identical devices with low variability and high yield. The device dimensions are longer L_ch_=567 nm, L_tg_=434 nm to avoid gate trench etch loading effects (Sect. 2.10). The yield criterion is I_max_/I_min_>10^5^ for a top gate sweep with fixed V_bg_=0 V, and Fig. 24 shows 5 typical wafers where all devices match this yield criterion. These 5 wafers have slight variations in the top gate stack deposition process without adversely impacting the yield.

### Device variability

#### Lot-to-lot and wafer-to-wafer variability

The lot-to-lot and wafer-to-wafer variability is tracked for the baseline flow with monolithic WS_2_ channel. This is done to detect drifts in the process caused by unintended tool or recipe changes. These wafers also act as reference wafers for experiments on e.g. the channel and gate stack processes.

**Fig. 25:**
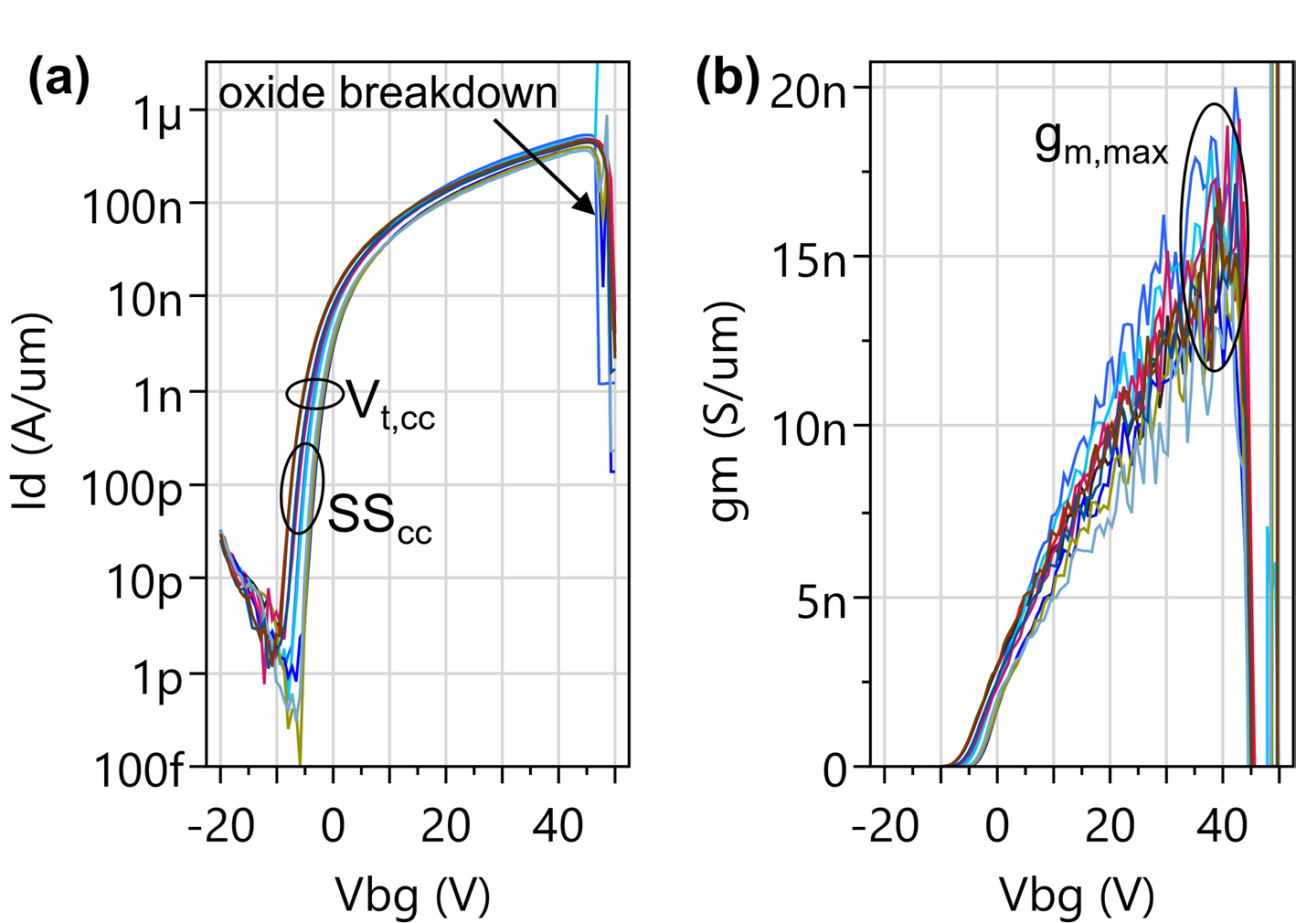
(**a**) Transfer characteristics of WS_2_-channel FETs with global back gate EOT = 50 nm, L_ch_=10 μm, W_ch_=1 μm at V_d_=1 V and extraction of the quality control parameters V_t, cc_ and SS_cc_. (**b**) No g_m_ peak is observed. Therefore, g_m, max_ is extracted close to oxide breakdown, from which µ_2PP, BD_ is calculated

The tracking is done using a long-channel reference device with L_ch_=10.1 μm, W_ch_=1 μm and global back gate configuration, from which three quality control parameters are extracted. Figure 25(a) shows the transfer characteristics for this reference device and the extraction of the three parameters:

- The threshold voltage at constant current of 1 nA/µm (**V**_**t, cc**_) gives a measure of (unintentional) doping by charged defects in the channel or the dielectric environment.
- The subthreshold swing at constant current of 100 pA/µm (**SS**_**cc**_) reflects the defect concentration with midgap energy level.
- Maximum transconductance (g_m, max_) normalized with the channel width, which is then converted to the parameter **µ**_**2PP, BD**_ using the formula µ_2PP, BD_ = g_m, max_ * L_ch_/(C_ox_*V_d_). This parameter is related to the field effect mobility.

The “best-effort” parameter µ_2PP, BD_ is defined since the 4-point-probe test structures (Sect. 3.2) were not present in older lots. Additionally, for monolithic WS_2_ which has a poor quality, we are unable to extract the mobility using TLM or the field effect mobility method which are commonly used in 2D FET literature [31]. Both methods require a g_m_ peak to be present in the linear regime, but Fig. 25(b) shows g_m_ keeps increasing at higher V_bg_ until the global back gate oxide breaks down. TCAD simulations [28] suggest the absence of the g_m_ peak is caused by an exponentially increasing trap density at higher energy levels close to the conduction band edge, to the point that the trap density exceeds the free charge concentration even in the linear regime. This causes stretch-out of the transfer characteristics and suppresses the g_m_ peak within the measurable back gate voltage range. Therefore, the parameter µ_2PP, BD_ is calculated using the same formula as the field effect mobility but takes the highest measurable g_m_max_ instead of g_m, peak_. and is a lower limit for the field effect mobility.

**Fig. 26:**
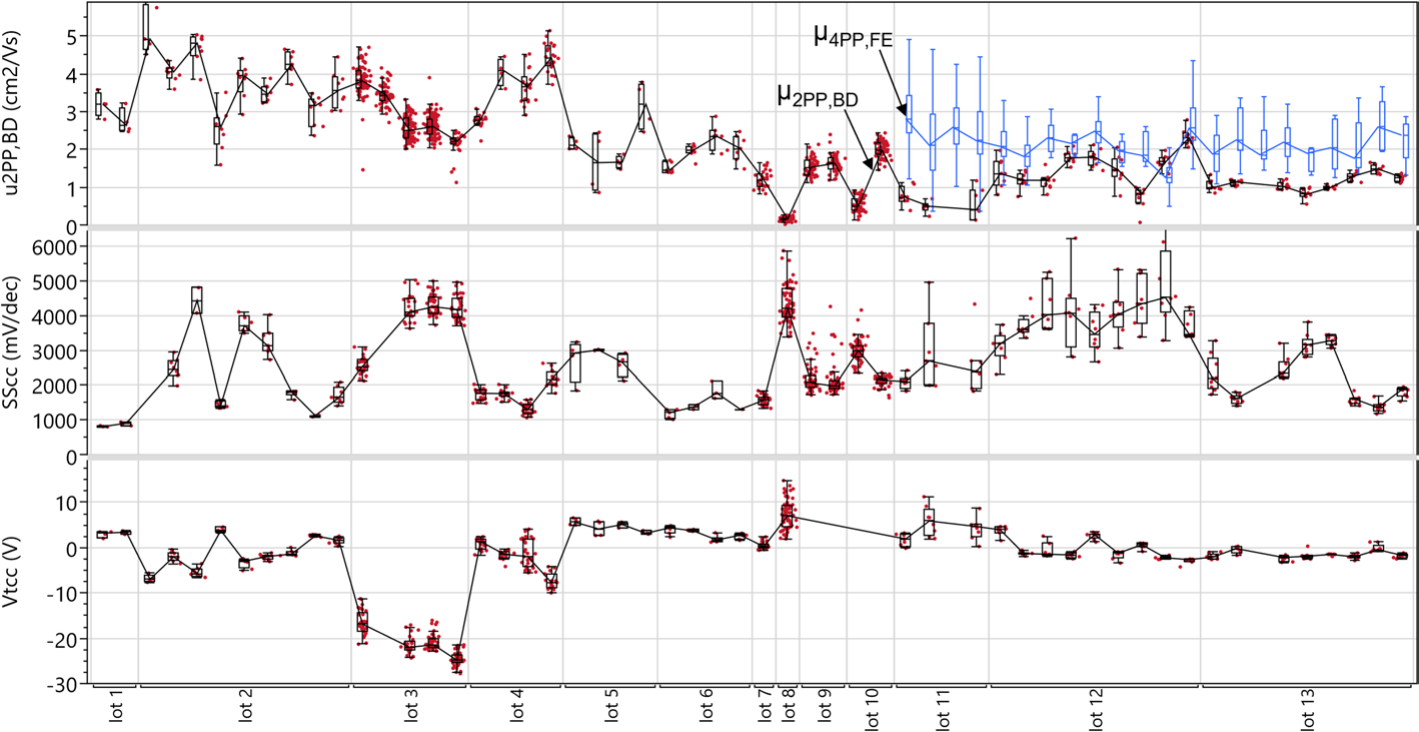
Systematic tracking of key quality control parameters for reference device with global back gate and L_ch_=10.1 μm, W_ch_=1 μm

The variability of the quality parameters µ_2PP, BD_, SS_cc_, V_t, cc_ from reference devices with global back gate and L_ch_=10.1 μm, W_ch_=1 μm is shown in Fig. 26. A first observation is that the within-wafer variability is small compared to wafer-to-wafer and lot-to-lot variability. The parameter µ_2PP, BD_, which represents a lower limit for the channel mobility, hovers around 3 cm^2^/V∙s but drops to 1 cm^2^/V∙s for the latest lots. Therefore we augment the plot with the parameter µ_4PP, FE_ from 4-point test structures with L_ch_=1 μm, W_ch_=0.3 μm, which are described in Sect. 3.2. µ_4PP, FE_ does not suffer from contact-induced mobility underestimation, and the values are systematically higher and more stable than µ_2PP, BD_, indicating that even the devices with long L_ch_=10.1 μm suffer from a contact resistance penalty, which can be in the range of 100kΩ-µm. The parameter SS_cc_ (representing mid-gap defectivity) has a typical range 1–2 V/dec but can go as high as 5 V/dec for some wafers (which is poor even considering the EOT = 50 nm). The parameter V_t, cc_ (representing unintentional n-doping) is typically around 0–5 V but goes to -25 V for some lots/wafers.

Multiple attempts have been made to quantitatively and qualitatively correlate these parameter fluctuations to physical measurements, but so far without success, as the main variability source(s) remain unknown. Possible candidates are fluctuations in defectivity of the channel, the interface passivation with the top dielectric or bulk defects in the dielectric, strain, or contact-induced effects. It is important to note that in the baseline flow is operated in a pilot line environment, where nearly all process steps have small fluctuations, either intended or unintended. The resulting wafer-to-wafer variability in the quality control parameters illustrates the high sensitivity of TMDC FET to these process fluctuations.

#### Device to device variability (single wafer)

**Fig. 27:**
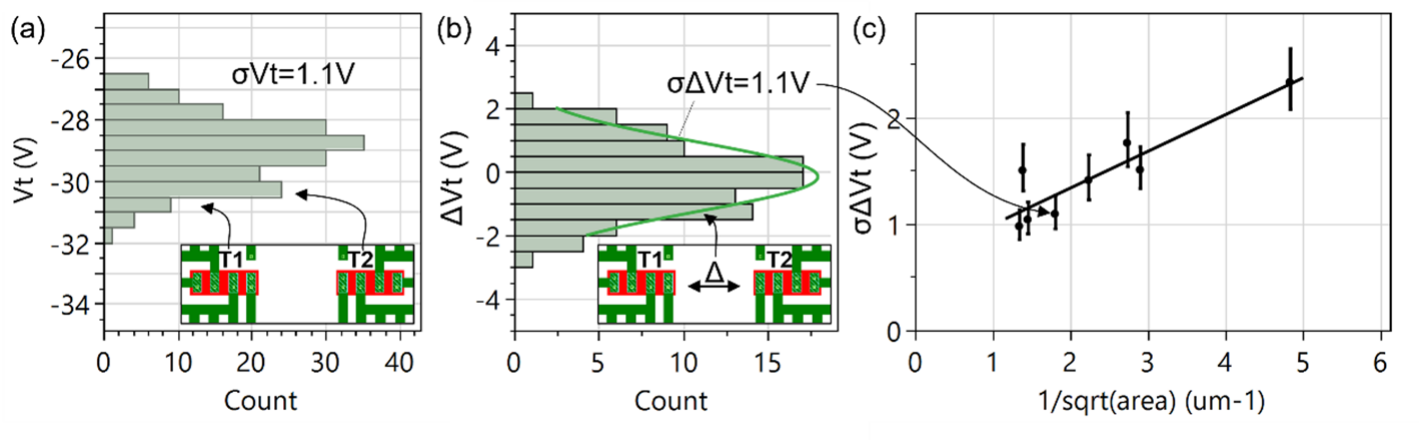
(a) The V_t_ distribution of transistors T1 and T2 with identical L_ch_=0.56 μm and W_ch_=1 μm over a full wafer is considered. The width of this distribution is exacerbated by center-to-edge process variations. (b) These long-range variations are eliminated with mismatch pair analysis, where the V_t_ difference between closely spaced transistors T1 and T2 are considered, resulting in smaller variance. (c) The σΔV_t_ are extracted for different device areas and mapped in a Pelgrom plot, which shows that larger channel areas cause random fluctuations to cancel out more

The device-to-device variability within a single wafer is relatively smaller. It is evaluated by extracting the standard deviation of the threshold voltage. Figure 27(a) shows an example of the V_t_ variability of WS_2_ FETs with the global back gate configuration with EOT = 50 nm, Al_2_O_3_ interlayer, W_ch_=0.5 μm, L_ch_=0.56 μm. The entire population of identical transistors T1 and T2 over a full wafer is fitted to a normal distribution, resulting in σV_t_=1.1 V, which is large due to the thick EOT = 50 nm, but also due to long-range nonuniformity across the wafer.

Examples of “extrinsic” variability sources caused by long-range nonuniformity (typically wafer center to edge), as observed by physical characterization methods :

- Average WS_2_ thickness (average density of bilayer islands).
- Average thickness of the AlOx interlayer by TMA soak method.
- SiO_2_ encapsulation thickness, causing variations in M0 trench depth and top gate trench depth.

Examples “intrinsic” variability sources caused by short-range nonuniformity (< 1 μm) that have been identified using SEM, TEM or other methods:

- Local WS_2_ thickness fluctuations (presence of bilayer islands).
- Point defects in the channel or gate stack.
- Local thickness fluctuations of the Al_2_O_3_ interlayer by TMA soak method.
- Nanocrystalline regions in top and/or bottom high-k layers.
- Variations in the gated width or length.

To cancel out long-range nonuniformity sources, mismatch pairs analysis is performed (Fig. 27(b)). Transistors T1 and T2 in the inset have identical dimensions and are closely spaced (typically 1 μm apart) and are measured simultaneously. The V_t_ difference between the two is extracted (ΔV_t_) to only probe the local difference between the two closely spaced devices. If the individual transistors in the pair would be uncorrelated and follow the global distribution, we would obtain σ(ΔV_t_)=$\:\sqrt{2}$σ(V_t_)=1.56 V (because the variances are additive). However we extract σ(ΔV_t_) = 1.1 V in Fig. 27(b), implying a 30% variability reduction by considering closely spaced pairs.

We construct a Pelgrom plot by repeating this procedure for transistor pairs with different dimensions and plotting σ(ΔV_t_) versus the inverse of the square root of the gated channel area in Fig. 27(c). We observe that the variability decreases for larger devices, as random fluctuations are cancelled out by the averaging effect. However, σ(ΔV_t_) does not go to zero in the limit of very large devices; the lines do not cross the origin. This can be interpreted as extrinsic variability sources not being fully cancelled out by the mismatch pairs. We evaluate the Pelgrom slope A_vt_=0.39 V∙µm, which is large due to the thick back gate EOT = 50 nm. Therefore, the same procedure is repeated with top gated devices with more scaled top EOT = 3 nm and we obtain much better A_vt_=18 mV∙µm. These two A_vt_ values for back and top gated 2D FETs form a baseline upon which to improve by reducing short-range variability sources listed above. To put these values into perspective, the lowest variability for 2D FETs was achieved on lab-scale back-gated devices, with monolayer MoS_2_ without second-layer islands and without capping, resulting in A_vt_=2.2 mV∙µm [33]. This strong reduction in A_vt_ for those lab devices compared to the current fab-compatible flow, is likely caused by (1) the more scaled back gate EOT, (2) the higher-quality MoS_2_ channels where bilayer islands were removed [33, 34], and (3) the absence of a top gate stack potentially inducing more disorder. For Silicon FETs, the state of the art is A_vt_=0.86 mV∙µm for TSMC 3 nm node [35].

### CET evaluation by C-V

**Fig. 28:**
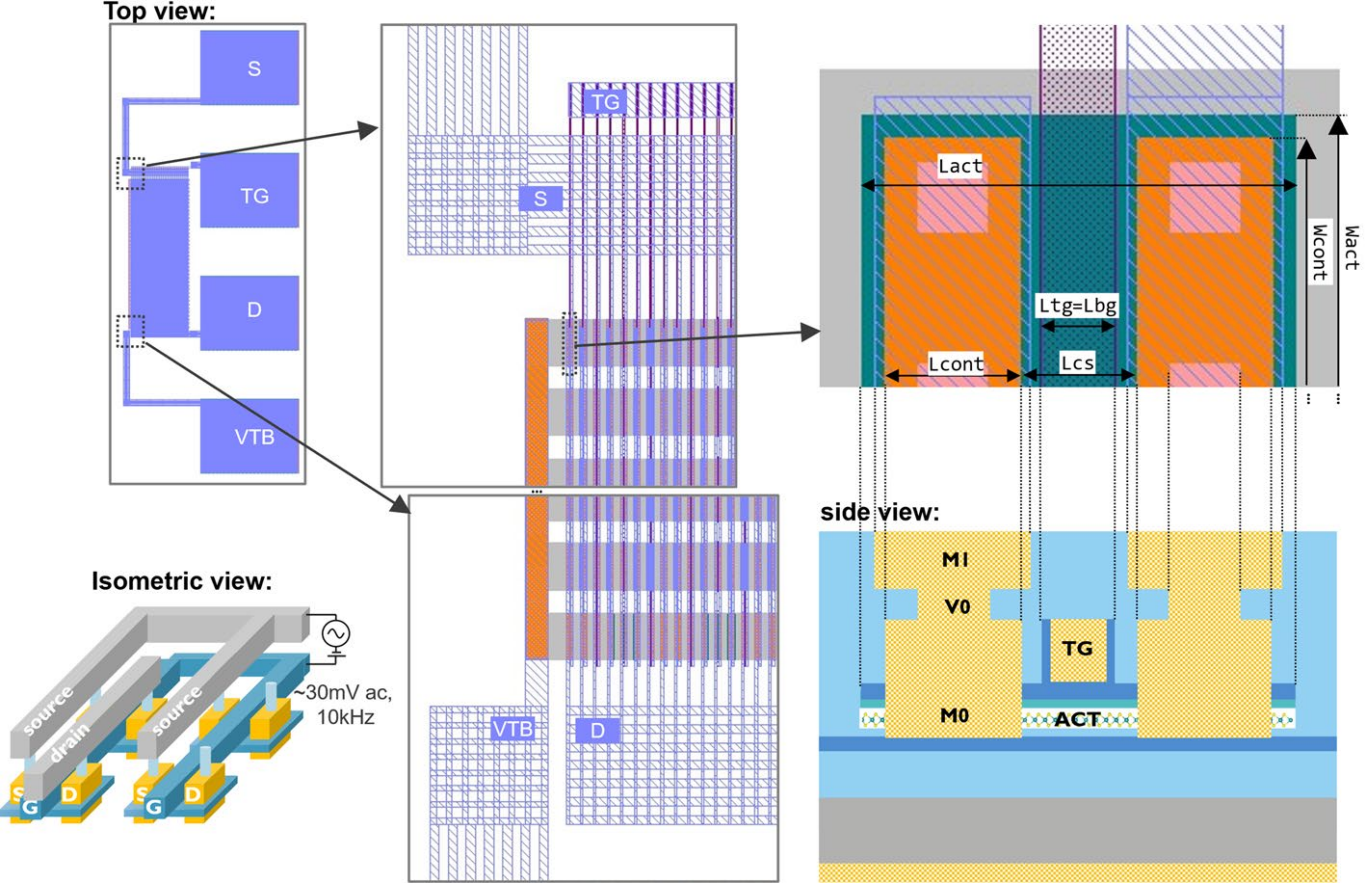
FETCAP design is distributed with 40 × 40 cells connected together at BGC, TG and M1 level. The isometric view shows a cut-out of only 4 cells

While the Effective Oxide Thickness (EOT) is typically extracted from the physical oxide thickness and the dielectric permittivity, the Capacitive Effective Thickness (CET) is measured using capacitance-voltage (C-V) measurements on dedicated test structures called FET capacitors (FETCAPs), which are shown in Fig. 28. Instead of a large single capacitor area, the design is distributed, and the cells are connected in parallel. This has the following benefits:

- Multiple cells connected in parallel boost the gated area and therefore the capacitance signal.
- W_ch_ and L_ch_ are kept relatively small (< 5 μm), complying with the design rules and ripout sensitivity (Supplementary Information Figure S5).
- Keeping L_ch_ sufficiently small (< 5 μm) limits the series resistance effect of long TMDC channels, which causes a drop in perceived capacitance, especially at high AC frequencies [36–38].

**Fig. 29:**
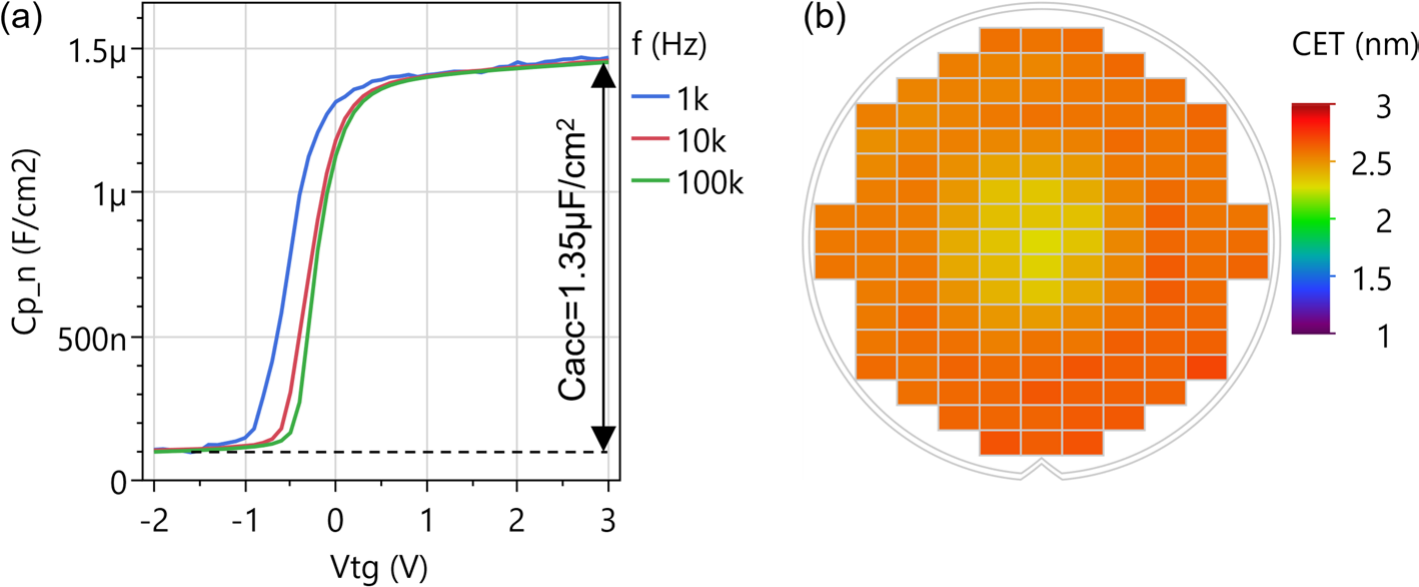
(**a**) C-V in a top-gated WS_2_ FET from depletion to accumulation at 3 frequencies. V_bg_=30 V is applied. (b) Mapping of the capacitive equivalent thickness across the 300 mm wafer

For n-type channels with a top gate EOT ~ 2.5–3 nm and a global back gate EOT ~ 50 nm, the measurement is typically as follows: The source and drain voltages are set to 0 V and the global back gate is set to a constant voltage of 30 V to accumulate electrons in the access regions. The top gate DC voltage range is chosen − 2:3 V to sweep across the depletion and accumulation regions of the channel. The AC small signal amplitude is set to 30 mV and the frequencies are set to 1 kHz, 10 kHz, 100 kHz. An example FETCAP measurement is shown in Fig. 29(a) for a top-gated WS_2_ FET with 1.5 nm AlO_x_ interlayer, 5 nm HfO_2_ cap and 3 nm HfO_2_ top-up in the gate trench. The FETCAPs have L_tg_=500 nm (mask dimension), W_ch_=2 μm, with 40 × 40 cells.

The equivalent parallel capacitance C_p, n_ is obtained by dividing the measured capacitance (Cp) by the total gated area

$$\:{C}_{p,n}=\frac{{C}_{p}}{{W}_{ch,calib}\:{L}_{tg,calib}\:{n}_{f}\:{n}_{p}}$$.

where W_ch, calib_ and L_tg, calib_ are the TEM-calibrated channel width and top gate length, n_f_ is the number of fingers and n_p_ is the number of lines in the FETCAP design (both 40 for this example). The accumulation capacitance (C_acc_) is calculated as the difference between maximum and minimum capacitance $\:{C}_{p,n,max}-{C}_{p,n,min}$. The CET is calculated as

$$\:CET=\frac{{\epsilon\:}_{0}\:{\epsilon\:}_{SiO2}}{{C}_{acc}}$$.

The extraction of the accumulation capacitance C_acc_, and the mapping of the CET across a 300 mm wafer with WS_2_ FETs are shown in Fig. 29(a, b). The CET is 2.6 nm towards the wafer edge and mid-radius, and 2.4 nm in the wafer center. This is within the bracket for the expected EOT = 2–2.8 nm, calculated from the dielectric constant k = 6 ± 2 for the 1.5 nm Al_2_O_3_ interlayer, k = 21 for 3 nm HfO_2_ cap and 3 nm HfO_2_ top-up, the EOT range being caused by the uncertainty on the dielectric constant. The slightly thinner CET in the wafer center is attributed to a deeper overetch into the HfO_2_ cap during the top gate trench etch, seen in TEM images. Apart from this center-to-edge nonuniformity, the low CET variability demonstrates excellent process control for the interlayer and cap deposition, the top gate trench etch and the high yield for the 1600 cells connected in parallel.

### Device reliability

**Fig. 30:**
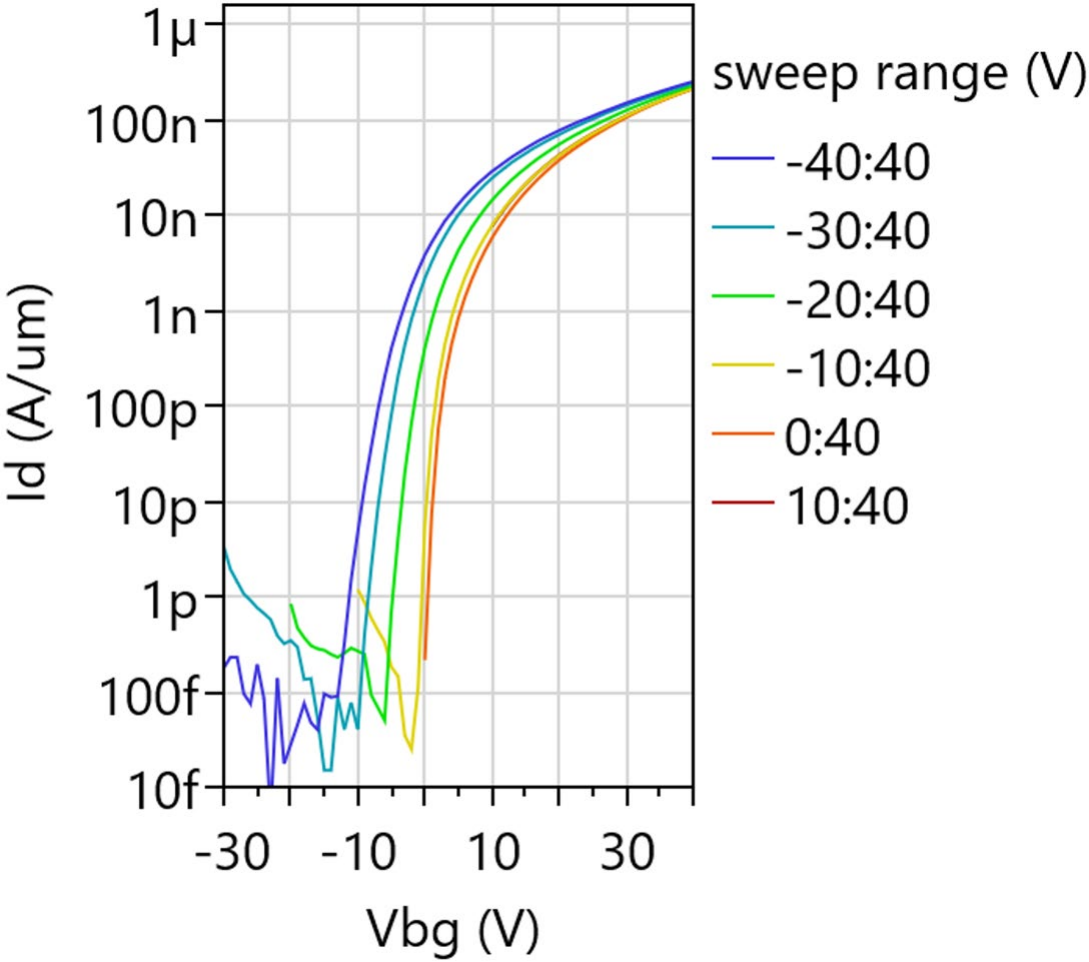
Transfer characteristics of global back gate devices with monolithic WS_2_ are sensitive to the chosen sweep range. Starting the sweep from more negative voltages causes more negative V_t_ due to trapping of positive charges in the gate oxide(s). W_ch_=20 μm, L_ch_=10 μm, V_d_=1 V

Device reliability is typically evaluated in terms of degradation or threshold voltage (V_t_) shifts under electrical stress. Such shifts have direct long-term detrimental implications, as they progressively reduce gate overdrive at a fixed operating bias, leading to degraded drive current, increased switching delay, and reduced noise margins. For the baseline process with WS_2_ channel (Sect. 2.5.1) with an AlO_x_ interlayer (Sect. 2.7), significant reliability challenges remain. These issues are most evident in the pronounced hysteresis and the strong dependence on the sweep starting voltage, as illustrated in Fig. 30. When the sweep begins at moderate voltages (10, 0, or − 10 V), the results are nearly indistinguishable. However, if the sweep starts from more negative voltages (–20, − 30, − 40 V), including the device off-state, V_t_ shifts strongly in the negative direction and the subthreshold swing degrades. This behavior is attributed to negative bias temperature instability (NBTI), which arises from the trapping of positive charges in the gate oxide or at the interfaces during the initial part of the sweep, leading to a negative V_t_ shift. Conversely, positive bias temperature instability (PBTI) occurs when a positive gate stress induces a positive V_t_ shift. NBTI and PBTI are also responsible for the hysteresis observed in the transfer characteristics.

**Fig. 31:**
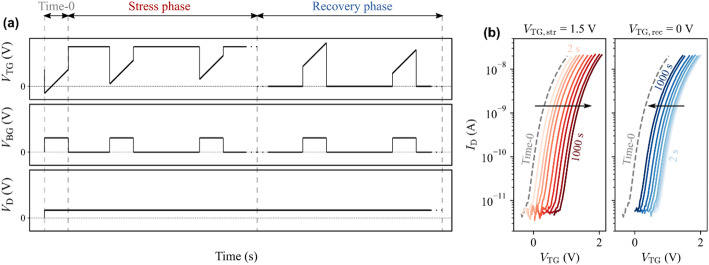
(**a**) BTI extraction using the measure–stress–measure (MSM) method adapted for top gate + global back gate devices. (**b**) Representative results showing that applying a top gate bias stress (V_TG, str_ = 1.5 V) for stress times from 2 s to 1 ks results in progressively larger positive V_t_ shifts (PBTI). The top gated device has L_tg_=2 μm, W_ch_=1 μm

We quantify NBTI and PBTI using the measure–stress–measure (MSM) methodology previously reported [14]. Figure 31(a) illustrates the MSM procedure applied to top-gate and global back-gate devices. First, the “time-0” top-gate and/or back-gate V_t_​ values are obtained using a narrow voltage sweep to avoid trap charging. A stress voltage is then applied for 2 s, after which the corresponding transfer characteristics are measured again using an adjusted voltage range to capture the V_t_​ shift. This cycle is repeated with exponentially increasing stress times up to 1 ks. Device relaxation is subsequently assessed by removing the applied stress and tracking the recovery of the V_t_​ shift. Figure 31(b) shows representative top-gate sweeps during PBTI stress and recovery, where progressive positive V_t_​ shifts arise from the trapping of negative charges (stress), and negative V_t_​ shifts occur due to their emission (recovery). Our previous work demonstrated that most of the trapping occurs in the AlO_x_ interlayer [39]. This behavior originates from the high defect density introduced during deposition and from the traps’ unfavorable energetic alignment just below the conduction band minimum of WS_2_.

**Fig. 32:**
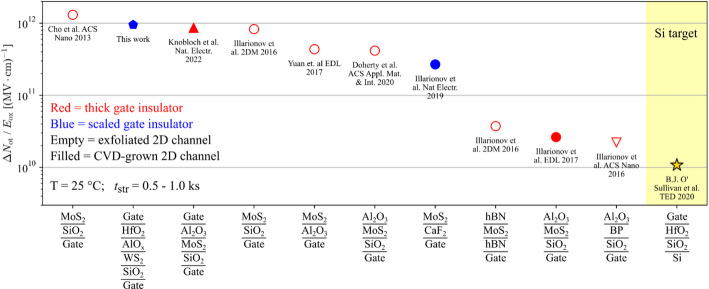
Benchmark plot of ΔN_ot_​/E_ox_. The stability of our 300 mm-integrated WS_2_​ FETs is poor compared to other 2D FET prototypes reported in the literature. Major improvements in device processing and/or alternative channel–oxide material combinations are required to drastically reduce the density of active traps

After converting the measured ΔV_t_​ into the corresponding variation in trapped charge concentration projected to the channel interface (ΔN_ot_​) [14], device stability is evaluated. Specifically, ΔN_ot_​ normalized by the stress electric field (at room temperature after 1 ks stress) is benchmarked against other 2D FET prototypes reported in the literature (Fig. 32) [40–48]. Significant advances in device processing and/or alternative channel–dielectric material combinations are still required to minimize active traps and meet the reliability standards established for Si technologies. Although several dielectrics compatible with 2D TMDs—such as CaF₂ and native high-*k* oxides—have demonstrated low charge trapping and promising electrical performance [16, 45, 49], their compatibility with standard semiconductor fabrication processes remains an open challenge.

### Device stability

Device stability or ageing is understood as the degradation of performance with time, even under nominal storage conditions. After initial electrical characterization of all EOL lots, the processed wafers are stored in clear front-opening shipping boxes (FOSB) under ambient environment. The stability of the fabricated devices is then monitored over time by remeasuring wafers after certain periods in storage to verify any changes in their performance parameters. New measurements are always performed on “fresh” devices with identical dimensions to avoid BTI effects.

**Fig. 33:**
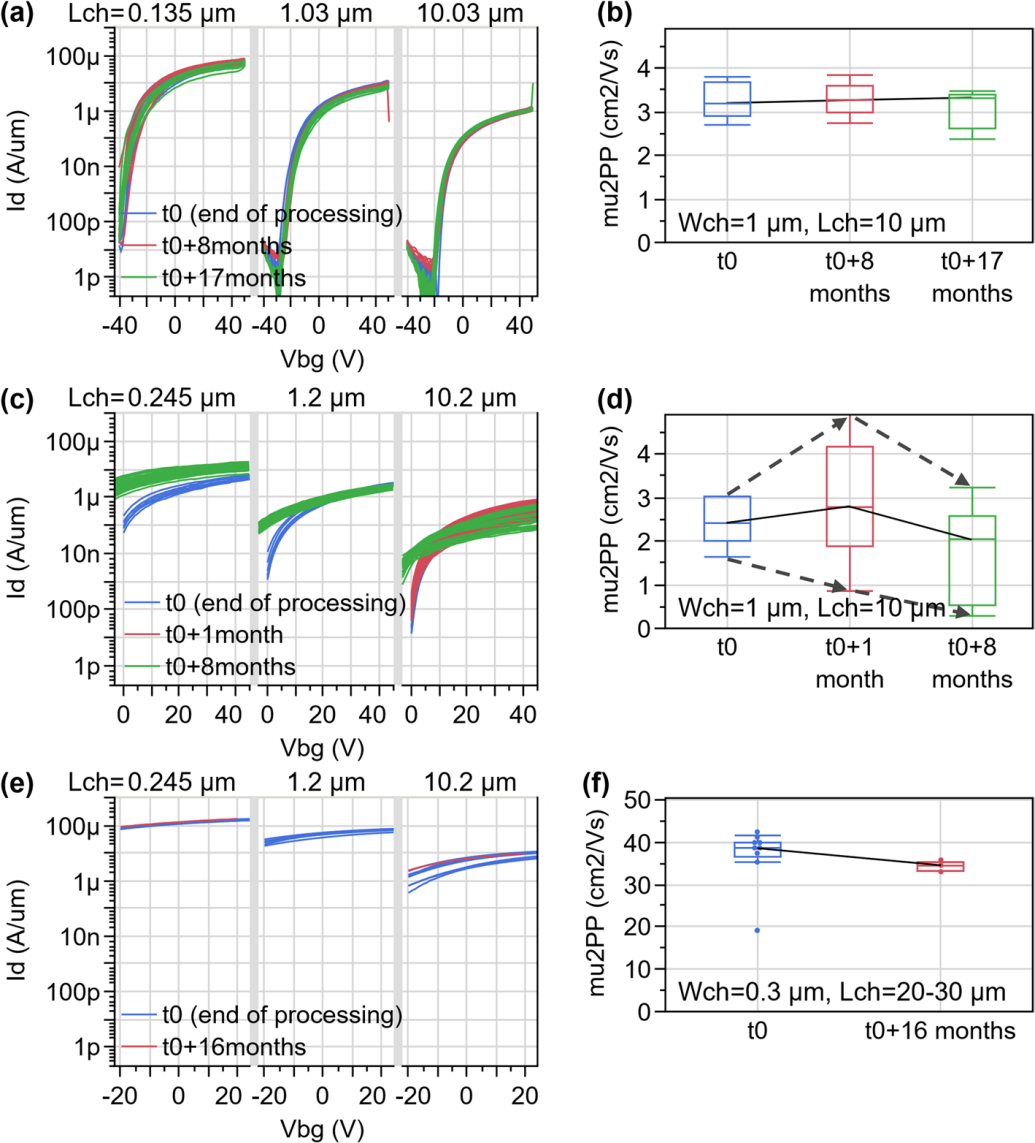
The transfer characteristics and 2-point field effect mobility of global back gated FETs are repeatedly measured over time. (**a**-**b**) A first lot with WS_2_ channels shows stable characteristics with time. (c-d) A second lot with WS_2_ channels shows severe degradation of the modulation. (**c**) A third lot with transferred MoS_2_ channels shows stable characteristics with time. All indicated times are after the end of processing

The transfer characteristics in Fig. 33 show some lots are stable with time, while others are plagued by ageing. The evaluated devices have the global back gate configuration and the channel material is either monolithic WS_2_ for the first two lots in Fig. 33(a-d) or transferred MoS_2_ for the case of Fig. 33(e-f). All channels are capped by Al_2_O_3_/HfO_2_ and ~ 300 nm SiO_2_ encapsulation, with only M1 metals remaining exposed to the storage environment. For the first lot with WS_2_ channels, the devices are stable 17 months after the end of processing. The transfer characteristics and 2-point field-effect mobility display no significant alteration.

In contrast, devices from the second lot with WS_2_ channel in Fig. 33(c-d) exhibit a negative V_t_ shift and a loss of current modulation after 8 months, with these effects being more pronounced in devices with channel lengths below 1 μm. Long-channel (10 μm) devices show slightly higher mobility and increased variability after only one month of storage, which then degrades when reaching the 8-month period.

Interestingly, devices from the third lot with MoS_2_ channel in Fig. 33(e-f), showed minimal signs of these effects, with only a small degradation on long-channel mobility after 16 months of storage. The strong n-doping of MoS_2_ discussed in Sect. 3.3.2 is not a result of ageing. It is difficult to relate this effect to the fundamental intrinsic properties of the materials used, since they are grown and processed in different ways. It is speculated that direct-grown WS_2_ (on SiO_2_) could be more affected due to its polycrystalline characteristics and higher defect density, while transferred MoS_2_ grown epitaxially on sapphire presents substantially lower defect density and no extended grain boundaries. This possibly process-related ageing effect is not yet understood, and currently under investigation.

## Conclusions

A research vehicle was developed to study planar FETs with monolayer TMDC channels. The flow is compatible with and entirely executed in a 300 mm pilot line. The efforts and limitations towards the obtention and processing integration of high-quality 2D channels, either monolithic deposition of WS_2_ over a full wafer, or templated growth of MoS_2_ on sapphire are highlighted. The transfer techniques employed enable the decoupling of material growth and fabrication, which is complemented by a plasma treatment to remove polymer residues and to recover the pristine TMDC surface. Additionally, different device configurations are demonstrated and extensively characterized. Global back gated devices display the highest yield (> 99%) due to the lowest fabrication complexity. Despite their simplicity, they are best suited for channel and contact studies. Top-gated devices enable gate stack and EOT scaling studies, enabled by the improved TMA soak process for the deposition of a closed and uniform interlayer and high-k dielectric stack across the entire wafer area. Nonetheless, improvements and alternatives are demanded to reduce the reliability issues caused by carrier trapping in this interlayer. Additionally, improvements are needed to address the device stability issues encountered. The ability to perform the fabrication in a 300 mm Si pilot line also allows a variability analysis across lots, within wafers, and with mismatch pairs. The results described here provide a foundation for further improvements of the devices and processes, enabling future advancements in channel quality, gate stack engineering, and variability. In the future, the fab-compatible device vehicle presented in this manuscript will require further improvements and refinements to be technologically relevant. Most importantly, work is needed on further reduction of the contact resistance, on controllable doping, and a better understanding of the processes impact on device performance. Also, for ultra scaled planar devices, a module for self-aligned gate to source-drain is required. Finally, for more advance architectures such as FinFETs or gate-all-around (GAA) nanosheet FET, some modules will need to be adapted or entirely developed, such as sacrificial layer replacement, and GAA conformal deposition of the gate-stack.

## Supplementary Information

Below is the link to the electronic supplementary material.

Supplementary material 1

## Data Availability

The datasets generated during and/or analysed during the current study are available from the corresponding author on reasonable request.
