# Supplementary material for: Integration and electrical evaluation of WS2 and MoS2 fets in a 300 mm pilot line
Source: Discov Electron. 2026 Feb 1;3(1):15. doi: 10.1007/s44291-026-00164-4 (PMC12862033; doi:10.1007/s44291-026-00164-4)
Supplement: Supplementary file 1 — Supplementary material 1 [file 44291_2026_164_MOESM1_ESM.docx]

# Supplementary information

## Local back gate integration


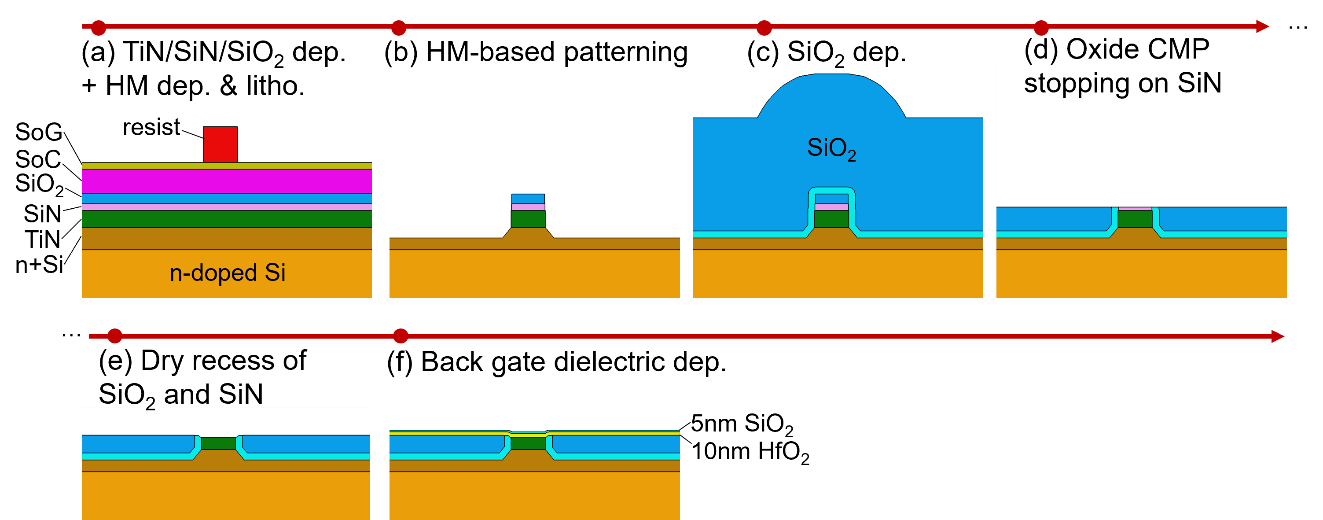


Figure S1: The process steps to fabricate the TiN local back gate. (a) Low-roughness TiN deposition and SiN/SiO_2_/SoC/SoG mask deposition and lithography. (b) TiN patterning, while retaining the SiN and some of the SiO_2_. (c) SiO_2_ deposition and (d) planarization CMP stopping on the SiN. (e) Uniform SiO_2_ and SiN etch back. (f) Deposition of back gate dielectric stack.

Figure S1 shows the COVENTOR-simulated local back gate process module, where the local back gate is made of TiN. The local back gate can also be made of Si, by modifying step (a) where TiN is omitted and step (b) where the Si local back gate is etched in the substrate. The original Si or TiN surface smoothness is protected by the SiN, preventing damage and scratching by CMP.

## Process steps for active patterning


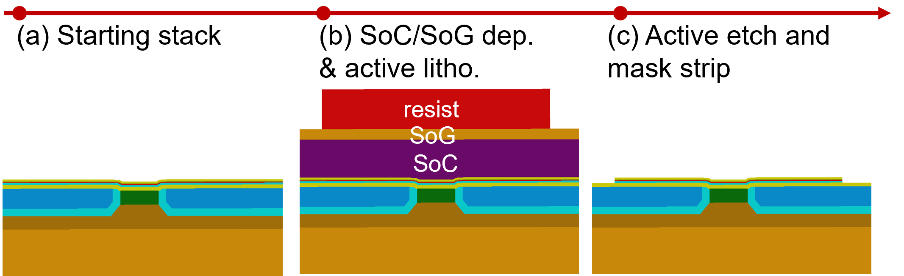


Figure S2: The process steps for active area patterning. (a) The starting stack has a TiN local back gate electrode and back gate dielectric stack, the TMDC channel, the interlayer and HfO_2_ cap layers. (b) Soft mask coating (Spin on Carbon + Spin on Glass), resist and lithography. (c) Pattering of the HfO_2_ cap, interlayer, and the underlying TMDC channel in a single etch step, followed by SOC mask strip in oxygen plasma.

## Process steps for the M0 (contact) module


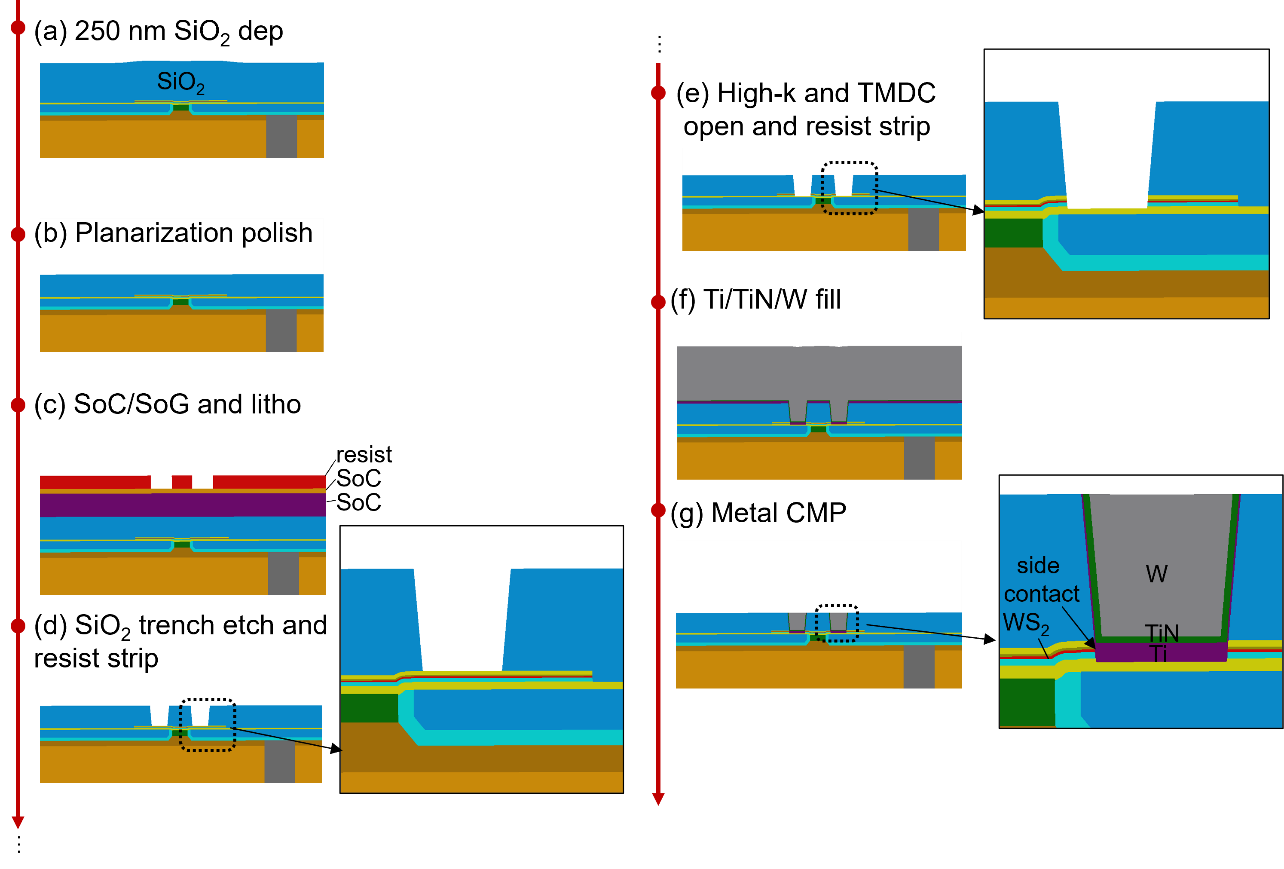


Figure S3: The process steps for the M0 side contact formation. (a) 250 nm SiO_2_ deposition. (b) Planarization polish keeping ~100 nm SiO_2_. (c) SoC/SoG deposition and lithography. (d) SiO_2_ trench etch stopping on the HfO_2_ cap, followed by resist strip. (e) HfO_2_ cap, interlayer and TMDC etch. (f) Ti (PVD) / TiN (ALD) / W (ALD+CVD) deposition. (g) Metal CMP.

## Process steps for the top gate module


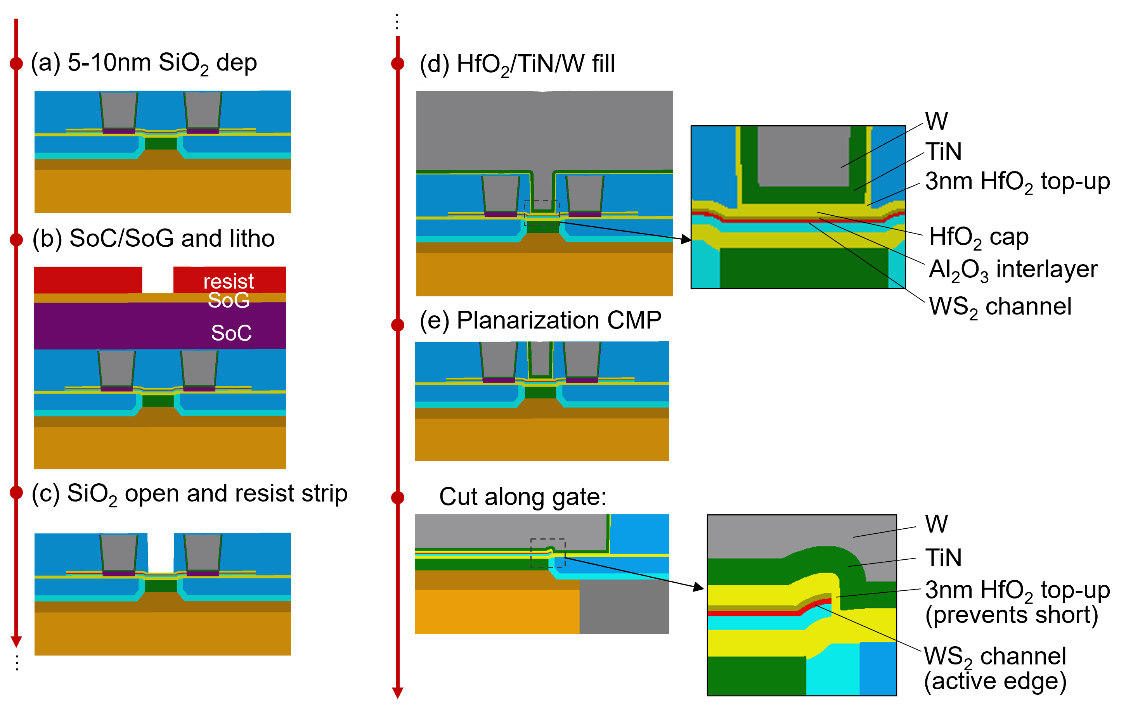


Figure S4: The process steps for the top gate formation. (a) 5-10 nm SiO_2_ deposition to allow litho rework. (b) SoC/SoG deposition and lithography. (c) SiO_2_ trench etch stopping on the HfO_2_ cap, followed by resist strip. (d) Deposition of 3 nm HfO_2_ by ALD, TiN by ALD, W fill by ALD CVD in gate trench. (e) Metal CMP.


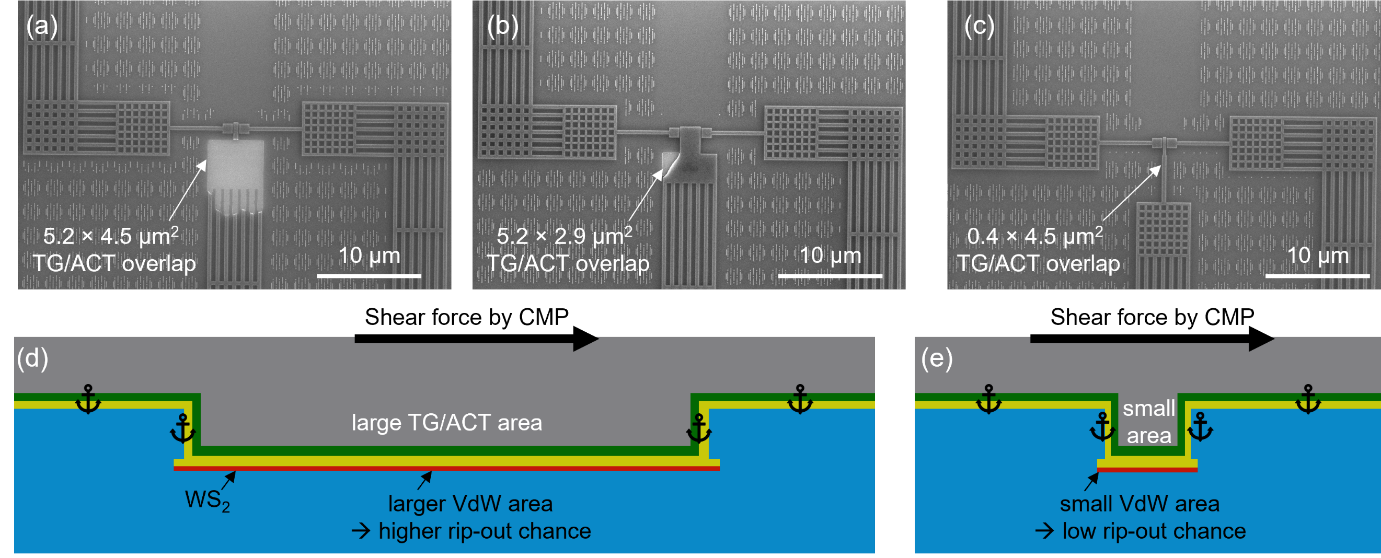


Figure S5: (a) Gate rip-out is observed after top gate metal CMP where the TG/active overlap areas are large. (b) Partial delamination for intermediate areas. (c) No delamination for small areas. (d) Schematic cross section during top gate CMP explaining that large TG/active overlap areas have relatively fewer anchoring surfaces to redistribute the shear stress to the surrounding oxide, causing delamination at the vander Waals interface (stack not drawn to scale). (e) Small TG/active overlap areas have relatively more anchoring, and are therefore less prone to delamination.

During the W CMP, systematic top gate rip-out is observed for where the overlap area of top gate on active area is larger than 4 × 4 µm^2^ squares, as illustrated in Figure S5(a-c). Top gate rip-out also occurs in regions with a gridded top gate design on non-gridded active underneath. This gate rip out can be explained by the considerable shear forces acting on the weak TMDC interfaces. Figure S5(d) shows only the sidewalls and SiO_2_-to-SiO_2_ interfaces surrounding the devices provide anchor points that can redistribute the shear force. Figure S5(e) shows a smaller TG/ACT overlap area has relatively more anchoring and is therefore less prone to rip-out.

## Process steps for Via To Bottom


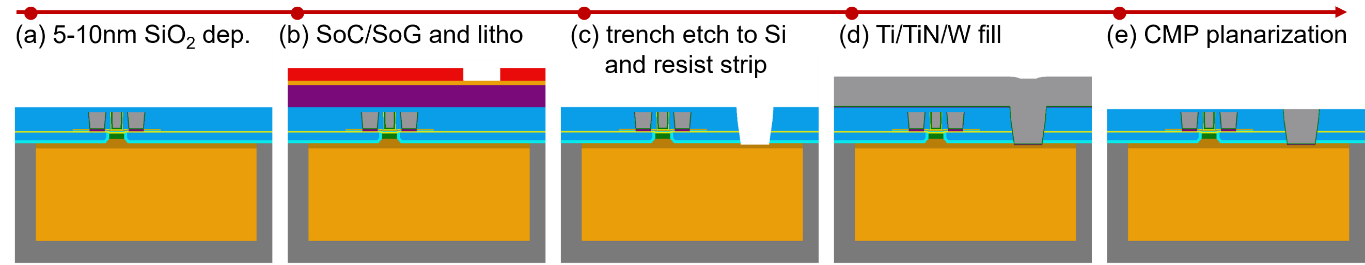


Figure S6: The process steps for the Via To Bottom connection, which ultimately connects the BGC to a front side pad. (a) 5-10 nm SiO_2_ deposition to allow litho rework. (b) SoC/SoG deposition and litho. (c) Trench etch stopping in the doped Si BGC, followed by resist strip.(d) Ti/TiN/W fill. The Ti is used as a contact to the n+ Si BGC. (e) Metal CMP.

## Process steps for W-based V0 and M1


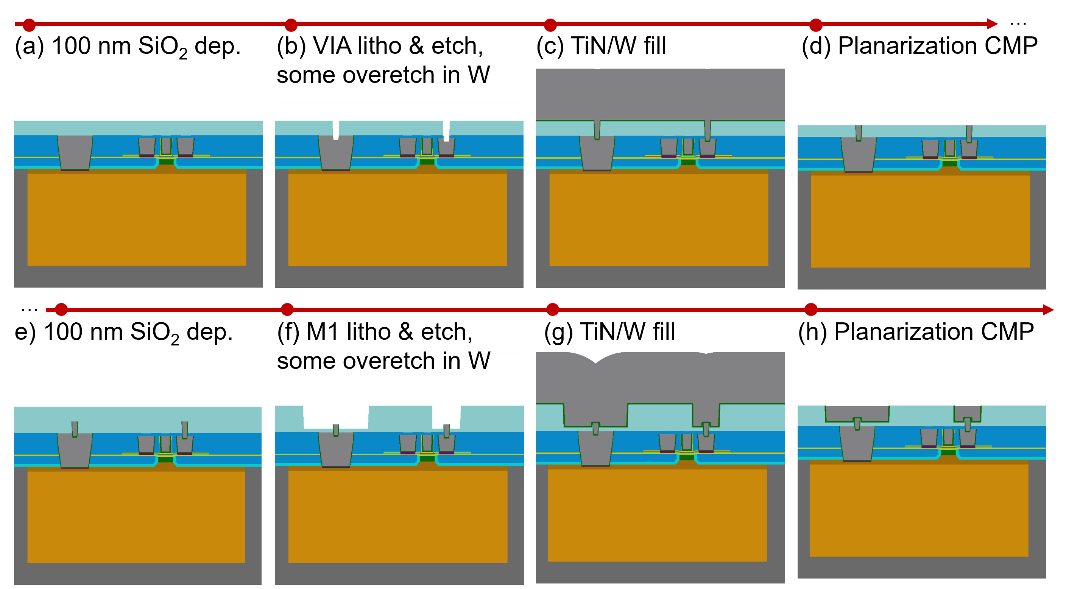


Figure S7: The process steps for the damascene VIA and M1 process are very similar. (a,e) Deposition of SiO_2_ at reduced temperature. (b,f) SiO_2_ etch with some over-etch on the W to remove the WO_x_ layer. (c,g) Deposition of TiN liner by ALD and W by ALD and CVD. (d,h) Metal CMP.

## Key figures of merit

| **Channel** | **1.1L WS_2_ (monolithic)** | | | **1.2L MoS_2_ (templated)** |
| --- | --- | --- | --- | --- |
| **Configuration** | **GBG** | **TG+GBG** | **LBG** | **GBG** |
| Gate stack | 50 nm SiO2 | 1.5 nm Al_2_O_3_/ 5+3 nm HfO_2_ | 10 nm HfO_2_/ 5 nm SiO_2_ | 50 |
| Target EOT (nm) | 50 | 2.2-2.8 (top) | 7 | 50 |
| Measured CET (nm) | - | 2.6 |  |  |
| FE mobility (cm^2^V^-1^s^-1^) | 2.2 ± 0.8 (4PP) | - | - | 20-54 (2PP) |
| Yield | >99 | >99.8 | >11 | 97-99 |
| I_max_ (µA/µm) @Lch | 14.5 | 7.8 | 2.9 | 250 |
| SS (mV/dec) | 2915 | 113 | 810 | 460 @ large area NA @ short L_ch_ |
| I_max_/I_min_ | 10^6^ | 10^5^ | 10^3^ | 10^8^ @ large area 2 @ short L_ch_ |
| 2Rc (kΩ∙µm) | 10-100 | - | - | 5 |
| Variability A_vt_ (mV∙µm) | 390 | 18 | - | - |
| BTI trapped charge (proj.) ΔNot/Eox (MV∙cm)^-1^ | - | 10^12^  @1 ks stress | - | - |
| Stability | sometimes strong ageing | sometimes strong ageing |  | Little ageing |

Table S1: Overview of key figure of merits extracted for different channel materials and gate configurations throughout this article. Comparison of metrics between device architectures metrics should not be generalized and must be treated with utmost caution, as they are often strongly affected by processing non-idealities.
